# Fulvalene as a platform for the synthesis of a dimetallic dysprosocenium single-molecule magnet†
†Electronic supplementary information (ESI) available: Synthesis details, spectroscopic characterization, X-ray crystallography, magnetic measurements and computational details. CCDC 1993006, 1993008 and 1993009. For ESI and crystallographic data in CIF or other electronic format see DOI: 10.1039/d0sc02033h


**DOI:** 10.1039/d0sc02033h

**Published:** 2020-05-18

**Authors:** Mian He, Fu-Sheng Guo, Jinkui Tang, Akseli Mansikkamäki, Richard A. Layfield

**Affiliations:** a Department of Chemistry , School of Life Sciences , University of Sussex , Brighton , BN1 9QR , UK . Email: R.Layfield@sussex.ac.uk; b Changchun Institute of Applied Chemistry , Chinese Academy of Sciences , Renmin Street 5626 , 130022 Changchun , China . Email: tang@ciac.ac.cn; c NMR Research Unit , University of Oulu , P.O. Box 8000, FI-90014 , Finland . Email: Akseli.Mansikkamaki@oulu.fi

## Abstract

The dinucleating fulvalenyl ligand [1,1′,3,3′-(C_5_^*t*^Bu_2_H_2_)_2_]^2–^ (Fv^tttt^) was used to synthesize the dimetallic dysprosocenium cation [{Dy(η^5^-Cp*)}_2_(μ-BH_4_)(η^5^:η^5^-Fv^tttt^)]^+^ (**3**) as the salt of [B(C_6_F_5_)_4_]^–^ (Cp* = C_5_Me_5_). Compound [**3**][B(C_6_F_5_)_4_] was obtained using a method in which the double half-sandwich complex [{Dy(BH_4_)_2_(THF)}_2_(Fv^tttt^)] (**1**) was reacted with KCp* to give the double metallocene [{Dy(Cp*)(μ-BH_4_)}_2_(Fv^tttt^)] (**2**), followed by removal of a bridging borohydride ligand upon addition of [(Et_3_Si)_2_(μ-H)][B(C_6_F_5_)_4_]. The dimetallic fulvalenyl complexes **1–3** give rise to single-molecule magnet (SMM) behaviour in zero applied field, with the effective energy barriers of 154(15) cm^–1^, 252(4) cm^–1^ and 384(18) cm^–1^, respectively, revealing a significant improvement in performance across the series. The magnetic properties are interpreted with the aid of *ab initio* calculations, which show substantial increases in the axiality of the crystal field from **1** to **2** to **3** as a consequence of the increasingly dominant role of the Fv^tttt^ and Cp* ligands, with the barrier height and hysteresis properties being attenuated by the equatorial borohydride ligands. The experimental and theoretical results described in this study furnish a blueprint for the design and synthesis of poly-cationic dysprosocenium SMMs with properties that may surpass those of benchmark systems.

## Abstract 2

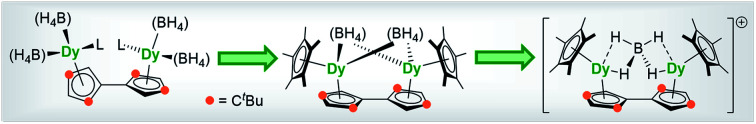
A series of fulvalene-supported dimetallic dysprosium metallocene SMMs provides a roadmap to poly-cationic dysprosocenium single-molecule magnets.

## Introduction

Observations of magnetic bistability in structurally well-defined, monodisperse nanomaterials have stimulated considerable interest in technology based on the quantum properties of atoms and molecules. For example, the demonstration of magnetic memory in single holmium atoms on surfaces has introduced potential for the fabrication of data storage devices with capacities surpassing those of conventional technology.^1^,^2^ Molecule-based magnetic materials offer similar opportunities, accompanied by the advantage that their electronic structure can be tuned using imaginative synthetic chemistry. Recent advances include magnetic molecules incorporated into spintronic devices or used as the basis of quantum algorithm operations.^3^–^5^

The magnetic hysteresis properties of single-molecule magnets (SMMs) have also been acclaimed as a possible source of novel data storage materials and, while such applications may eventually be possible, certain obstacles must first be overcome.^6^–^10^ Challenges to the implementation of SMM-based technology include that: (1) all known systems require cooling with cryogens in order to show hysteresis; (2) uniform nano-structuring of SMMs on surfaces is difficult, and; (3) the chemical stability of SMMs throughout surface deposition processes and, subsequently, in a device environment is not guaranteed, regardless of whether or not the bulk material itself is air-sensitive. However, encouraging progress has been made, such as the discovery of magnetic hysteresis in an SMM at 80 K,^11^*i.e.* above the boiling point of liquid nitrogen, and elegant surface studies of some SMMs have demonstrated the single-molecule origins of the hysteresis.^12^

The advances made to date strengthen the motivation for further research into SMMs, particularly the exploration of new synthetic strategies that aim to increase the temperatures at which these materials function. Considerable effort has been invested into maximizing the effective energy barrier to reversal of the magnetization (*U*_eff_) and the magnetic blocking temperature (*T*_B_). A few highly anisotropic metal ions have proven to be important as the basic ingredient in an SMM, with dysprosium being the most popular and terbium, erbium and cobalt(ii) also playing prominent roles.^13^–^21^ In the case of Dy^3+^, a 4f^9^ ion with oblate spheroidal electron density, successful SMM synthesis strategies tend to produce compounds in which the metal occupies a coordination environment with a strong and highly axial crystal field.^22^–^24^ This approach partly explains why the highest-performing SMMs are based on metallocene cations of the type [Dy(η^5^-Cp^R^)_2_]^+^, where the bulky cyclopentadienyl ligands [Cp^R^]^–^ provide the strong, axial crystal field whilst also blocking the formation of any deleterious equatorial crystal field.^6^,^11^,^25^–^29^

Previous work on sandwich SMMs constructed with erbium-COT (COT = cyclo-octatetraenyl)^30^,^31^ and terbium-phthalocyanine^32^,^33^ building blocks has shown that the parameters *U*_eff_ and *T*_B_ increase when two or more blocks are linked to form multi-decker sandwich complexes. We therefore reasoned that improvements in the SMM properties of dysprosocenium cations might be possible if closely related polymetallic versions could be synthesized. To investigate this idea, we targeted the synthesis of a dimetallic dysprosocenium cation by replacing a cyclopentadienyl ligand with a bicyclopentadienyl ligand, also known as fulvalenyl or pentafulvalenyl, in which two cyclopentadienyl rings share an exocyclic carbon–carbon bond.^34^ Dinucleating fulvalenyl ligands have been used extensively in transition metal sandwich chemistry,^35^ with recent examples including macrocyclic poly(ferrocenyl) compounds with potential applications as molecular electronic materials.^36^,^37^ In contrast, the use of such ligands in f-element chemistry is uncommon, and in the case of the lanthanides was, hitherto, limited to the dimetallic divalent compounds [M(THF)(η^5^:η^5^-Fv^tttt^)]_2_ with M = Sm, Eu or Yb.^38^

## Results and discussion

**Scheme 1:**
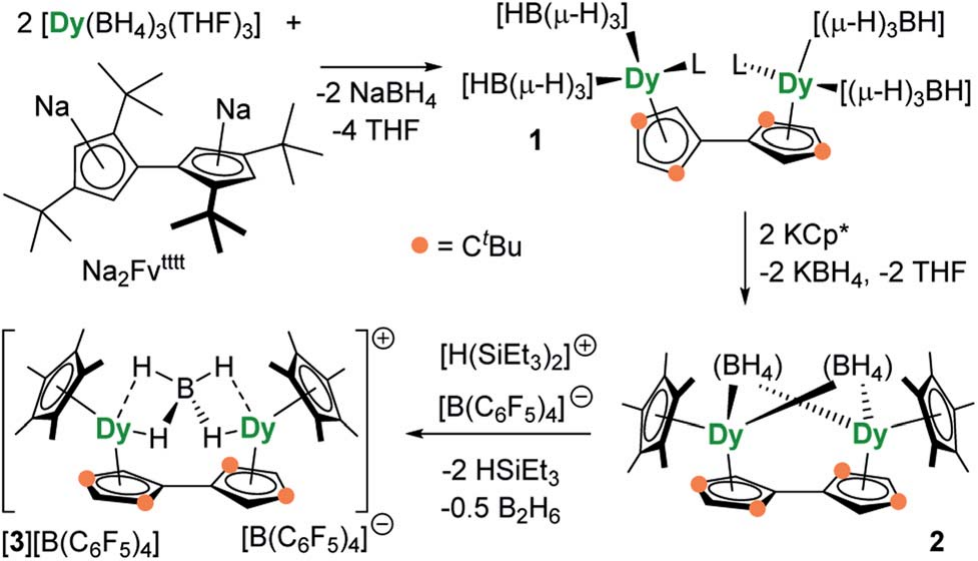
Synthesis of compounds **1**, **2** and [**3**][B(C_6_F_5_)_4_] (L = THF).

The reaction of the di-sodium salt of 1,1′,3,3′-tetra-*tert*-butylpentafulvalenyl, Na_2_Fv^tttt^, with two equivalents of [Dy(BH_4_)_3_(THF)_3_] produced the dimetallic complex [{Dy(BH_4_)_2_(THF)}_2_(η^5^:η^5^-Fv^tttt^)] (**1**), with the two half-sandwich units linked *via* the fulvalenyl ligand, as shown in Scheme 1. Subsequently, compound **1** was reacted with two equivalents of potassium pentamethylcyclopentadienide (KCp*) to give the double metallocene [{Dy(η^5^-Cp*)(μ-BH_4_)}_2_(η^5^:η^5^-Fv^tttt^)] (**2**) in which the dysprosium centres are bridged by both borohydride ligands. Finally, addition of one equivalent of the super-electrophile [(Et_3_Si)_2_(μ-H)][B(C_6_F_5_)_4_] to compound **2** produced the separated ion-pair [{Dy(η^5^-Cp*)}_2_(μ-BH_4_)(η^5^:η^5^-Fv^tttt^)][B(C_6_F_5_)_4_] ([**3**][B(C_6_F_5_)_4_]), containing the target dimetallic dysprosocenium cation **3**, with the two sandwich units bridged by a single borohydride ligand.

**Fig. 1:**
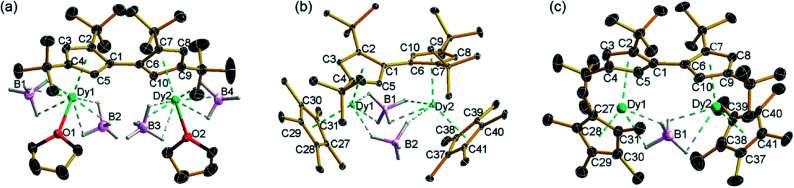
Thermal ellipsoid representations (30% probability) of the structures of: (a) **1**, (b) **2** and (c) **3**. For clarity, hydrogen atoms are omitted except those in the [BH_4_]^–^ ligands.

The molecular structures of all three compounds were determined by single-crystal X-ray diffraction (Fig. 1 and Table S1†). The structure of **1** consists of two similar half-sandwich units in which each dysprosium is ligated by one cyclopentadienyl unit of the Fv^tttt^ ligand in an η^5^-manner, with additional coordination by two κ^3^-borohydride ligands and a THF ligand (Fig. 1). The Dy1–C distances are in the range 2.607(4)–2.698(4) Å and the distance from Dy1 to the fulvalenyl C_5_ centroid is 2.361(1) Å; the analogous distances for Dy2 are essentially the same (Table S2†). Appreciable twisting of the two halves of the molecule about the central Fv^tttt^ carbon–carbon bond is evident from the C2–C1–C6–C7 torsional angle of 95.3(6)°, resulting in an intramolecular Dy···Dy separation of 5.443(1) Å. Diagnostic absorptions in the FTIR spectrum of compound **1** occur at *υ̃* = 2127–2467 cm^–1^ for the terminal and bridging B–H groups (Fig. S1†).

In compound **2**, the two unique halves of the molecule each consist of a dysprosium centre sandwiched between an η^5^-Cp* ligand and an η^5^-Fv^tttt^ ligand, with bridges between the metal atoms occurring *via* both borohydride ligands. The change in the bonding mode of the borohydride ligands in **2** as compared to those in **1** is presumably a consequence of the spatial demands of the Cp* ligands. For Dy1, the bond distances to the Cp* ligand are in the range 2.643(3)–2.667(2) Å and those to the fulvalenyl ligand are 2.630(2)–2.704(4) Å, with associated Dy1–centroid distances of 2.362(1) Å and 2.378(1) Å, respectively; the corresponding distances involving Dy2 are not significantly different (Table S3†). The metallocene bending angles subtended at Dy1 and Dy2 are 137.913(1)° and 139.143(1)°, respectively. The bridging borohydride ligands in **2** greatly reduce the extent of twisting between the two halves of the Fv^tttt^ ligand relative to **1**, as shown in the C2–C1–C6–C7 torsional angle of 30.0(4)°. The Dy···Dy separation is, at 4.148(1) Å, also markedly shorter than that in **1**. The IR absorptions for the borohydride ligands occur in the range *υ̃* = 2124–2473 cm^–1^ (Fig. S2†).

In the structure of compound **3**, the bonding modes of the Cp*, Fv^tttt^ and borohydride ligands are similar to those in **2**. Thus, the Dy1–C distances to the Cp* and Fv^tttt^ ligands are in the range 2.594(7)–2.650(8) Å and 2.578(7)–2.742(8) Å, respectively, with associated Dy1–centroid distances of 2.348(1) Å and 2.355(1) Å, respectively. As in compounds **1** and **2**, the geometric parameters for Dy2 are similar to those of Dy1 in compound **3** (Table S4†). However, the two metallocene components of **3** are markedly less bent than those in **2**, with bending angles of 145.727(1)° and 146.246(1)° for Dy1 and Dy2, respectively. The fulvalene twist angle is 55.0(9)° and the Dy···Dy separation is 4.701(1) Å. The borohydride ligand adopts a κ^2^:κ^2^ bonding mode, with associated IR frequencies occurring as broad absorptions around *υ̃* = 2230 cm^–1^ (Fig. S3†).

The synthesis of **1**, **2** and [**3**][B(C_6_F_5_)_4_] demonstrates that fulvalenyl ligands can indeed form the structural basis of linked dysprosium metallocenes. In the next stage, our aim was to investigate how the changes in the crystal field environment experienced by the Dy^3+^ centres impact upon the SMM properties.

### Magnetic properties

The magnetic properties of **1**, **2** and [**3**][B(C_6_F_5_)_4_] were studied in static (DC) and dynamic (AC) magnetic fields using a Magnetic Property Measurement System. The temperature dependence of the product of the molar magnetic susceptibility and temperature, *i.e. χ*_M_*T*(*T*), is consistent with the dimetallic composition of the three compounds. The values of *χ*_M_*T* at 300 K are 27.3, 27.2 and 27.5 cm^3^ K mol^–1^ for **1**, **2** and [**3**][B(C_6_F_5_)_4_], respectively, are close to the theoretical value of 28.2 cm^3^ K mol^–1^ for a complex containing two weakly interacting Dy^3+^ ions with ^6^H_15/2_ ground multiplets (Fig. S4, S6 and S8†).^39^ The decreases in *χ*_M_*T* with temperature for **1** and [**3**][B(C_6_F_5_)_4_] are similar and gradual without featuring a sharp drop at lower temperatures, reaching values of 22.2 and 21.9 cm^3^ K mol^–1^ at 2.0 K. The decrease in *χ*_M_*T* with temperature for **2** is similar in the high-temperature regime, however a much more pronounced decrease was observed below 20 K such that a value of 17.5 cm^3^ K mol^–1^ is reached at 2.0 K. The decrease in *χ*_M_*T*(*T*) with decreasing temperature for all three compounds can be accounted for by gradual depopulation of the excited crystal field levels of the Dy^3+^ ions, which may occur concomitantly with antiferromagnetic exchange coupling becoming more prominent as thermal randomization effects diminish, particularly in compound **2**. The isothermal field-dependence of the magnetization, *M*(*H*), for each compound also reflects the presence of two Dy^3+^ ions per complex, reaching values of 9.89, 10.11 and 9.60 *Nβ* at 7 T and 1.9 K for **1**, **2** and [**3**][B(C_6_F_5_)_4_], respectively (Fig. S5, S7 and S9†).

**Fig. 2:**
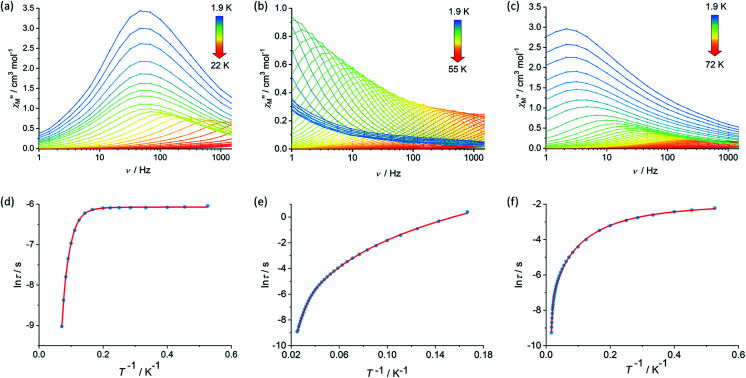
Frequency dependence of the imaginary component of the AC susceptibility, *χ*′′ *versus ν*, in zero DC field for (a) **1**, (b) **2** and (c) [**3**][B(C_6_F_5_)_4_] at the temperatures indicated, and the temperature dependence of the relaxation time as ln *τ versus T*^–1^ for **1** (d), **2** (e) and (f) [**3**][B(C_6_F_5_)_4_].

The real (*χ*′) and imaginary (*χ*′′) components of the AC magnetic susceptibility were measured for each compound as functions of temperature and AC frequency (*ν*) (Fig. 2, S10–S12, S15–S17, S20–S22†), using zero DC field and a small AC field of 3 Oe. The observation of well-defined maxima in the *χ*′′(*T*) and *χ*′′(*ν*) plots for **1**, **2** and [**3**][B(C_6_F_5_)_4_] indicate slow relaxation of the magnetization without the need for an external DC field. For compound **1**, maxima were observed in the *χ*′′(*ν*) plot from 1.9–14 K before the upper frequency limit of the measurement system is reached (Fig. 2). Up to 6 K, the position of the frequency maximum and, hence, the relaxation time (*τ*), varies only slightly with temperature, suggesting that relaxation *via* quantum tunnelling of the magnetization (QTM) is dominant in this regime. At higher temperatures, the frequency maximum becomes strongly temperature dependent, which is likely to reflect thermally activated relaxation becoming dominant. The relaxation times for each temperature were extracted and plotted as a function of *T*^–1^, revealing that the change from QTM to activated regimes is abrupt (Fig. 2). Fitting the data with the equation *τ*^–1^ = *τ*_0_^–1^e^–*U*_eff_/*k*_B_*T*^ + *CT*^*n*^ + *τ*_QTM_^–1^, where *τ*_0_^–1^ and *U*_eff_ denote the Orbach parameters, *C* and *n* denote the Raman parameters, and the rate of QTM is *τ*_QTM_^–1^, gives an energy barrier of *U*_eff_ = 154(15) cm^–1^ with *τ*_0_ = 3.93(6) × 10^–11^ s, the Raman parameters are *C* = 8.16(3) × 10^–4^ s^–1^ K^–*n*^, *n* = 5.87(1) and *τ*_QTM_ = 2.31(1) × 10^–3^ s.

The AC susceptibility measurements on compounds **2** and [**3**][B(C_6_F_5_)_4_] revealed that slow relaxation of the magnetization occur across wider temperatures ranges of 1.9–55 K and 1.9–72 K, respectively, relative to **1** (Fig. 2). In the case of **2**, the *χ*′′(*ν*) plot consists of well-defined maxima from 1.9 K up to 40 K, and for [**3**][B(C_6_F_5_)_4_] the maxima were observed from 1.9 K up to 60 K. The temperature dependence of the relaxation times for **2** revealed that the system does not cross to a purely temperature-independent regime even at the lowest measurement temperature (Fig. 2). Hence, it was possible to fit the ln *τ vs. T*^–1^ data for **2** without a QTM term, which produced Orbach parameters of *U*_eff_ = 252(4) cm^–1^, *τ*_0_ = 1.94(3) × 10^–8^ s and Raman parameters of *C* = 4.56 (2) × 10^–4^ s^–1^ K^–*n*^, *n* = 4.12(1). For [**3**][B(C_6_F_5_)_4_], after a strong dependence of ln *τ* on *T*^–1^ in the high-temperature regime, the relaxation time is only weakly temperature-dependent below 5 K. A fit of the data for this compound gave *U*_eff_ = 384(18) cm^–1^, *τ*_0_ = 1.37(6) × 10^–8^ s, *C* = 6.55(4) × 10^–1^ s^–1^ K^–*n*^, *n* = 2.03(2) and *τ*_QTM_ = 1.39 (6) × 10^–1^ s.

Comparing the *U*_eff_ parameters for **1**, **2** and [**3**][B(C_6_F_5_)_4_] reveals substantial increases on moving through the series. This trend can be understood in a qualitative sense by considering how the composition and geometry of the crystal field experienced by each Dy^3+^ centre varies. Whilst the Dy^3+^ ions in all three compounds must experience a dominant axial crystal field to show any slow relaxation properties in zero DC field, this is clearly relatively weak in **1** and stronger in **3**. The presence of only one cyclopentadienyl group per Dy^3+^ in **1** is sufficient to induce SMM behaviour by providing a relatively strong axial crystal field, but the two borohydride ligands and the THF ligand provide a competing equatorial field that limits the barrier height. In **2**, the additional [Cp*]^–^ ligands strengthen the axial component of the crystal field whilst simultaneously reducing the number of equatorial ligands, resulting in an increase in the barrier by approximately 100 cm^–1^ (60%) relative to **1**. Upon forming **3**, not only is another equatorial borohydride ligand removed from the coordination environment of both Dy^3+^ ions, the two metallocene units are also less bent by approximately 7°–8° and the individual cyclopentadienyl donor groups are slightly closer to the metal centres than in **2**. Consequently, the barrier height in **3** is approximately 130 cm^–1^ (50%) greater than that in **2**. Furthermore, the ln *τ versus T*^–1^ data are also consistent with the single-ion QTM being reduced by intramolecular exchange interactions between the Dy^3+^ ions. The Dy···Dy distances decrease in the order **1** > **3** > **2**, hence the QTM should be slowest in **2**, as observed.

Compounds **1**, **2** and [**3**][B(C_6_F_5_)_4_] further illustrate the magneto-structural correlation developed for dysprosium and terbium metallocene SMMs, in which the cyclopentadienyl ligands provide dominant axial crystal fields and the properties are attenuated by the equatorial ligands.^28^,^40^,^41^ In addition to accounting for the variation in energy barrier across the series, their hysteresis properties can also be interpreted in terms of the molecular structure. Thus, although the cyclopentadienyl ligands dominate the crystal field, the borohydride and THF ligands provide non-negligible equatorial components, which result in the magnetization *versus* field hysteresis occurring as narrow S-shaped curves at 1.9 K and various field in the range ±5 T with an average scan rate of 23 Oe s^–1^ for each compound (Fig. S14, S19 and S24†). There is essentially no hysteresis for **1**, however the hysteresis for **2** displays narrow loops centred on field values of approximately ±1.3 kOe and ±5.0 kOe, respectively, and [**3**][B(C_6_F_5_)_4_] shows a loop around ±1.7 kOe. These subtle effects are presumably a consequence of exchange bias between the Dy^3+^ centres affecting the rate of relaxation *via* QTM, suggesting that the borohydride ligands are effective at transmitting exchange interactions.^42^ This proposal is consistent with a recent study of magnetic exchange in [Ln(BH_4_)_3_] (Ln = Gd–Tm), in which it was found that {HBH} bridges between the metal centres allow antiferro- or ferro-magnetic exchange, albeit with very small coupling constants.^43^

### Theoretical analysis

**Fig. 3:**
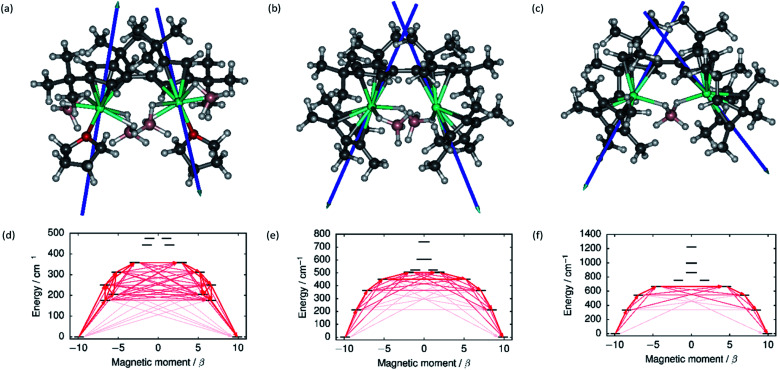
The principal magnetic axes of the ground KDs of the Dy^3+^ ions in (a) **1**, (b) **2** and (c) **3**. Calculated effective *ab initio* barriers for the relaxation of magnetization at the Dy1 ions (d) **1**, (e) **2** and (f) **3**. Stronger arrows indicate larger absolute value of the transition magnetic moment matrix elements between the respective states. Transitions involving higher-energy states not involved in the relaxation are omitted for clarity. The barriers for the Dy2 ions, which are similar to those of the Dy1 ions, are shown in ESI Fig. S25–S27.†

The local magnetic properties of each individual Dy^3+^ ion in **1–3** were first studied. State-averaged complete active space self-consistent field (SA-CASSCF) calculations^44^–^48^ followed by restricted active space state interaction treatment of spin–orbit coupling (SO-RASSI)^49^ and calculation of local magnetic properties using the SINGLE_ANISO_OPEN module^50^,^51^ were carried out on each ion while the other Dy^3+^ ion in the complex was replaced by diamagnetic yttrium. The properties of the eight lowest Kramers doublets (KDs) of each ion are listed in Tables S8–S13.† In each case the ground Kramers doublet (KD) is characterized by a strongly axial **g**-tensor with small but non-vanishing transverse components. The direction of the principal magnetic axis of each ground KD is determined by an axial-type interaction with the Fv^tttt^ and Cp* ligands (Fig. 3).

The first-excited local KDs of the two Dy^3+^ ions in **1** lie 176 cm^–1^ and 181 cm^–1^ above the ground KD. The angles between the principal magnetic axes of the ground and first excited states are 41.3° and 43.0°, respectively. These relatively large angles should lead to an efficient Orbach mechanism *via* the first-excited KD, and an effective barrier corresponding to the energies of the first excited states. Indeed, the effective barrier height determined from the fits is 154 cm^–1^ which agrees well with the calculated values. A qualitative *ab initio* barrier for the relaxation of magnetization was constructed using a well-established methodology,^52^ where the relaxation pathway is traced by following large magnitudes of transition magnetic moment matrix elements between different states. The barriers for **1** are shown in Fig. 3 and the quantitative transition magnetic moment matrix elements are listed in Table S14.† Based on the calculated values, the barrier should be crossed earliest at the first-excited KD and latest at the fifth-excited KD. Based on the experimental barrier height, the most likely relaxation takes place *via* the first-excited KD.

In **2**, the lowest four KDs on each Dy^3+^ ion have roughly collinear principal magnetic axes. The calculated transition magnetic moments (Fig. 3 and Table S15†) predict that the barrier becomes crossed either at the second- (364 cm^–1^ and 362 cm^–1^) or third-excited (452 cm^–1^ and 457 cm^–1^) local KDs. The experimentally determined barrier height (252 cm^–1^) is, however, closest to the energies of the first-excited local KD (214 cm^–1^ and 208 cm^–1^). The transition matrix elements for an Orbach process *via* the first excited KD are, however, not vanishingly small and this mechanism remains plausible. The first four KDs of Dy1 and the first three KDs of Dy2 in **3** are also roughly collinear. Based on the transition dipole moments (Fig. 3 and Table S16†) the barrier is most likely crossed at the second (546 cm^–1^ and 551 cm^–1^) or third excited KD (667 cm^–1^ and 666 cm^–1^). The experimentally determined barrier height (384 cm^–1^) is again closest to the energy of the first excited KD (336 cm^–1^ and 339 cm^–1^) and, again, an Orbach mechanism *via* the first excited KD has non-vanishing transition magnetic moment matrix elements and remains plausible.

Based on the energy of the first excited KD (which in all cases corresponds to the experimentally determined barrier height), the axiality of the crystal-field (CF) environments of the Dy^3+^ ions clearly increases from **1** to **3**. Further to the qualitative analysis of this trend (see above), quantitative insight was obtained by calculating the *ab initio* CF parameters^53^ for each Dy^3+^ ion and the results are listed in Tables S17–S19† using the Iwahara–Chibotaru notation.^54^ The effect of the CF can be understood qualitatively by considering the second-rank parameters, summarized in Table 1. The axial *B*_20_ parameters clearly increase from **1** to **2** to **3**, which is consistent with the increased axiality of the CF. The parameters should be compared to those calculated for the current benchmark SMM, *i.e.* the [(C_5_Me_5_)Dy(C_5_^i^Pr_5_)]^+^ cation, which has an energy barrier of 1540 cm^–1^ and a blocking temperature of 80 K,^11^ also listed in Table 1. It is immediately clear that the axiality falls short of that in [(C_5_Me_5_)Dy(C_5_^i^Pr_5_)]^+^ and the off-diagonal |*B*_2±2_| parameters of **1–3** are much larger. Thus, the equatorial borohydride ligands in **1–3**, and the lack of axial Cp* ligands in **1**, clearly lead to much reduced axiality.

**Table 1:** Rank two *ab initio* CF parameters (in cm^–1^) calculated for the Dy^3+^ ions in **1–3** and the [(C_5_Me_5_)Dy(C_5_^i^Pr_5_)]^+^ cation^11^ using Iwahara–Chibotaru notation

		*B*_20_	|*B*_2±1_|	|*B*_2±2_|
**1**	Dy1	–217.86	15.85	58.77
	Dy2	–221.95	18.45	56.36
**2**	Dy1	–375.17	18.19	81.70
	Dy2	–384.52	21.80	57.93
**3**	Dy1	–608.51	7.83	178.31
	Dy2	–615.72	8.76	203.13
[(C_5_Me_5_)Dy(C_5_^i^Pr_5_)]^+^		–1195.31	17.70	30.23

The non-negligible off-diagonal CF parameters lead to significant mixing of the different local angular momentum eigenstates defined by some definite angular momentum projection *M*. This is evident from Tables S20–S25,† which give the projections of the *ab initio* CF eigenstates on angular momentum eigenstates. The states in the ground KDs have a large contribution from a state with some definite *M*, but in all cases even the states in the ground KD are significantly mixed. This is consistent with the effective barrier being crossed at the first excited local KD in each case.

The effect of intramolecular interaction between the two Dy^3+^ ions was studied by the Lines model^55^ and the magnetic point-dipole approximation as implemented in the POLY_ANISO module.^51^,^56^,^57^ The Lines model describes the exchange interaction in terms of a single phenomenological scalar parameter which was determined by minimizing the standard deviation between the measured and calculated magnetic susceptibilities. Due to the general lack of low-temperature features in the susceptibility of **1** and **3** (Fig. S4, S8 and S28†), the parameter could only be only reliably determined for **2**. The eigenvalues of the Lines exchange operator and the dipolar coupling operator where mapped to an effective Ising-type Hamiltonian of the form:

1 *Ĥ* = –(*J*_ex_ + *J*_dipolar_)*S[combining tilde]*_z,1_*S[combining tilde]*_z,2_ = –*J*_tot_*S[combining tilde]*_z,1_*S[combining tilde]*_z,2_

In eqn (1), the *S[combining tilde]* = 1/2 pseudospin operators act on the ground KDs of the two Dy^3+^ ions. The dipolar coupling parameters *J*_dipolar_ are –0.9 cm^–1^, –2.5 cm^–1^ and –1.4 cm^–1^ for **1–3**, respectively, which are consistent with variations in the Dy···Dy distance between the two ions. The exchange parameter *J*_ex_ determined for **2** is –1.2 cm^–1^, hence the total exchange interaction is *J*_tot_ ≈ –3.7 cm^–1^. The absence of bridging borohydride ligands in **1** is likely to result in a vanishingly small *J*_ex_ parameter relative to **2**, which, combined with the smaller *J*_dipolar_ parameter, provides further support for the idea that the steps in the *M*(*H*) hysteresis in **2** and **3** occur as a consequence of exchange-bias effects.

## Conclusions

In developing a stepwise route from the fulvalenyl double half-sandwich complex [{Dy(BH_4_)_2_(THF)}_2_(Fv^tttt^)] (**1**) *via* the double metallocene [{Dy(η^5^-Cp*)(μ-BH_4_)}_2_(Fv^tttt^)] (**2**) to ion-separated [{Dy(η^5^-Cp*)}_2_(μ-BH_4_)(Fv^tttt^)][B(C_6_F_5_)_4_] ([**3**][B(C_6_F_5_)_4_]), we have shown that a dimetallic dysprosocenium complex can be synthesized. All three fulvalenyl-supported compounds are SMMs in zero applied field and show appreciable increases in the effective energy barrier to reversal of the magnetization across the series, which more than doubles from 154(15) cm^–1^ in **1** to 384(18) cm^–1^ in **3**. The improvements in the energy barrier are attributable to the increasingly dominant axial crystal field provided by the Fv^tttt^ and Cp* ligands balanced against the influence of the equatorial borohydride ligands. Quantitative support for this magneto-structural correlation was obtained from *ab initio* calculations, which revealed marked increases in the axial *B*_20_ parameter across the series but also with appreciable non-axial parameters.

It is instructive to consider these results in light of the SMM properties of [(C_5_Me_5_)Dy(C_5_^i^Pr_5_)]^+^ and its precursors, *i.e.* the monometallic metallocene [(C_5_Me_5_)Dy(C_5_^i^Pr_5_)(BH_4_)] and the half-sandwich [(C_5_^i^Pr_5_)Dy(BH_4_)_2_(THF)]. In the case of [(C_5_Me_5_)Dy(C_5_^i^Pr_5_)(BH_4_)], there many structural features in common with **2** and **3** and yet the energy barrier is a miniscule 7(1) cm^–1^ in zero field.^11^ This comparison further highlights how the strategy of combining metallocene building blocks to give a dimetallic dysprosocenium cation may improve SMM performance. In contrast, the barrier determined for [(C_5_^i^Pr_5_)Dy(BH_4_)_2_(THF)] in zero field is 241(7) cm^–1^, which is markedly larger than the barrier of 154(15) cm^–1^ in **1**. However, since the Dy–Cp_cent_ distances in the two half-sandwich complexes are very similar, the different barriers are likely to originate from the influence of the THF ligands. The Dy–O distances in **1** are approximately 0.03–0.08 Å shorter than those in its monometallic counterpart, pointing towards a stronger equatorial component of the crystal field and, therefore, relaxation *via* an Orbach process with a lower barrier.

Looking forward, if a dimetallic dicationic dysprosocenium complex such as [{Dy(η^5^-Cp*)}_2_(η^5^:η^5^-Fv^tttt^)]^2+^ could be synthesized, substantial increases in the axial crystal field parameters and decreases in the non-axial parameters should combine with the beneficial effects of exchange coupling in a dimetallic complex. Whilst substituent-dependent geometric factors will clearly play an important role in determining the properties, a dimetallic dysprosocenium dication can reasonably be anticipated to display enhancements in the energy barrier and blocking temperature relative to the current benchmark SMM.

## Conflicts of interest

There are no conflicts to declare.

## Supplementary Material

Supplementary information

Crystal structure data
