# Supplementary material for: Fulvalene as a platform for the synthesis of a dimetallic dysprosocenium single-molecule magnet
Source: Chem Sci. 2020 May 18;11(22):5745–52. doi: 10.1039/d0sc02033h (PMC7422961; doi:10.1039/d0sc02033h)
Supplement: Supplementary file 1 [file SC-011-D0SC02033H-s001.pdf]

Electronic Supplementary Material (ESI) for Chemical Science.
This journal is © The Royal Society of Chemistry 2020

Electronic Supplementary Information

Fulvalene as a platform for the synthesis of a dimetallic dysprosocenium single-molecule magnet

Mian He,[a] Fu-Sheng Guo,[a] Jinkui Tang,*[b] Akseli Mansikkamäki,*[c] Richard A. Layfield*[a]

[a] Department of Chemistry, School of Life Sciences, University of Sussex, Brighton, BN1 9QR, U.K.

[b] State Key Laboratory of Rare Earth Resource Utilization, Changchun Institute of Applied Chemistry, Chinese Academy of Sciences, Changchun 130022, P.R. China.

[c] NMR Research Unit, University of Oulu, P.O. Box 8000, FI-90014, Finland.

General synthetic procedures

All reactions were carried out under rigorous anaerobic and anhydrous conditions using argon or nitrogen atmospheres and standard Schlenk or glove-box techniques. Solvents were refluxed over an appropriate drying agent for a minimum of three days (molten potassium for toluene, THF, benzene-D_6, Na/K alloy for hexane) before being distilled, degassed and stored in ampoules over activated 4 Å molecular sieves. Glass-coated stirrer bars were used for each reaction. Elemental analyses were carried out at MEDAC Ltd. (Surrey, UK) or London Metropolitan University, U.K. IR spectra were collected on a Bruker Alpha FTIR spectrometer fitted with a Platinum ATR module. 1,1',3,3'-Tetra-*tert*-butyl-pentafulvalene ($(C_5{}^tBu_2H_3)_2$),[1] $[Dy(BH_4)_3(THF)_3]$,[2] and $[(Et_3Si)_2(\mu\text{-}H)][B(C_6F_5)_4]$[3] were prepared according to literature procedures.

Synthesis of $[\{Dy(BH_4)_2(THF)\}_2(Fv^{tttt})]$ (1)

Toluene (30 ml) was added to a mixture of 1,1',3,3'-tetra-*tert*-butyl-pentafulvalene (3.54 g,10.0 mmol) and $Na\{N(SiMe_3)_2\}_2$ (3.66 g, 20.0 mmol) cooled to 0°C. Then the reaction was stirred, warmed to room temperature and then heated to reflux overnight. The toluene was removed under vacuum and the residue washed with hexane (3 × 30 ml). A light-pink powder of $(NaC_5{}^tBu_2H_2)_2$ was obtained after filtration (1.75 g, 43 %), which can be isolated and used without further purification. Toluene was added to a mixture of $(NaC_5{}^tBu_2H_2)_2$ (1.00 g, 2.5 mmol) and $[Dy(BH_4)_3(THF)_3]$ (2.21 g, 5.3 mmol) at room temperature and the resulting suspension was stirred at 110°C for 48 hours. The solution colour changed from dark brown to pale orange/yellow. The solvent was removed under vacuum and the product was extracted into hexane (3 × 15 ml) and filtered. The solvent was removed slowly under vacuum until a crystal-like precipitate formed. Storage at –40 °C for two days produced pale-yellow crystals of **1**. The crystals were washed with cold hexane, re-dissolved in warm hexane and stored at –40 °C, which produced crystals of suitable quality for analysis by single-crystal X-ray diffraction. Isolated yield = 600 mg, 27 %.

Elemental analysis found (calcd.) % for $C_{34}H_{72}B_4O_2Dy_2$: C 46.14 (46.34); H 8.03 (8.24).
IR spectrum ($\tilde{\nu}/cm^{-1}$): 2956s, 2949s, 2899m, 2865w, 2467s, 2244w, 2206w, 2197m, 2127s, 1679w, 1463m, 1393w,1359m,1312w, 1238m, 1176s, 1097s,1056w, 1041w,1006s, 953m, 925m, 852s, 730w, 702m, 677m, 612w, 572w, 556w,509w, 434m.

Synthesis of $[\{Dy(Cp^*)(\mu\text{-}BH_4)\}_2(Fv^{tttt})]$ (2)

Solid **1** (500 mg, 0.6 mmol) was added in portions to a mixture of KCp* (199 mg, 1.1 mmol) and toluene (20 ml) at room temperature. The resulting suspension was refluxed for 48 hours, during which time the solution changed colour from yellow to orange. The toluene was removed under vacuum, and then the product was extracted into hexane (3 × 15 ml) and filtered. The solvent was concentrated slowly under vacuum to the point of incipient crystallization. Storing the resulting solution at –40 °C overnight produced colourless crystals of

2, which were re-crystallized to give bright-yellow single crystals of sufficient quality for analysis by X-ray diffraction. Yield = 200 mg, 36 %.

Elemental analysis found (calcd.) % for $C_{46}H_{78}B_2Dy_2$: C 56.25 (56.51); H 7.69 (8.04).
IR spectrum ($\tilde{\nu}$/cm^{-1}): 2956s, 2899s, 2861s, 2729w, 2473m, 2407w, 2228s, 2124m, 1608w, 1480w, 1457s, 1389m, 1361s, 1304w, 1265m, 1229s, 1198m, 1149s, 1092s, 1060m, 1022s, 959m, 918m, 850s, 826m, 806s, 697s, 669s, 613w, 594s, 552m, 514w, 432s.

Synthesis of [{Dy(η^5-Cp*)}$_2$(μ-BH$_4$)(η^5:η^5-Fv^{tttt})][B(C_6F_5)$_4$] ([3][B(C_6F_5)$_4$])
A solution of **2** (205 mg, 0.2 mmol) in hexane (10ml) was added to [$(Et_3Si)_2$(μ-H)][B(C_6F_5)$_4$] (191 mg, 0.2 mmol) at room temperature, which immediately produced a gel-like material. After stirring for 24 hours, a yellow powder had formed. The hexane was removed, the residue was washed with hexane (5 × 10 ml) and the resulting yellow powder dried under vacuum. Dissolving the powder in 1,2-dichlorobenzene (5 ml) and layering the solution with hexane at room temperature, produced, after several days, bright yellow crystals. The solvents were then decanted away and the crystals were washed with cold hexane (3 × 5 ml). The crystallization process was repeated twice in order to obtain single crystals of **3** suitable for X-ray diffraction. Yield = 100 mg, 29 %.

Elemental analysis found (calcd.) % for $C_{70}H_{74}B_2F_{20}Dy_2$: C 50.09 (51.21); H 4.32 (4.54).
IR spectrum ($\tilde{\nu}$/cm^{-1}): 2958m, 2929w, 2916w, 2870m, 2230w, 1642m, 1511s, 1462s, 1412w, 1394w, 1382w, 1367w, 1271s, 1236m, 1202w, 1080s, 1028w, 977s, 926w, 906w, 840s, 773s, 756s, 726w, 713w, 682s, 661s, 611m, 572m, 430w.

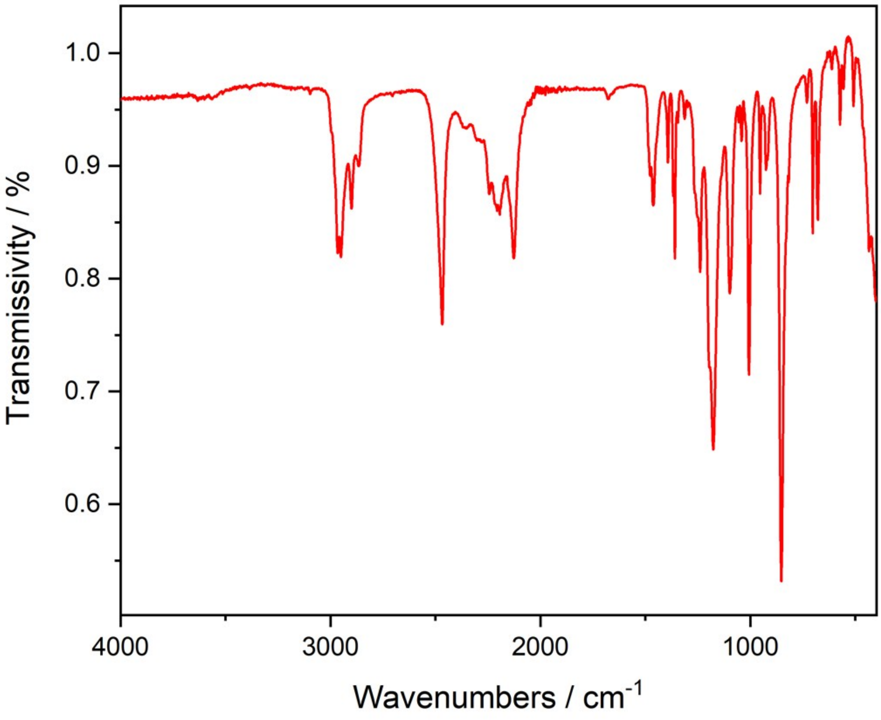

Fig. S1. Infrared spectrum of compound **1**.

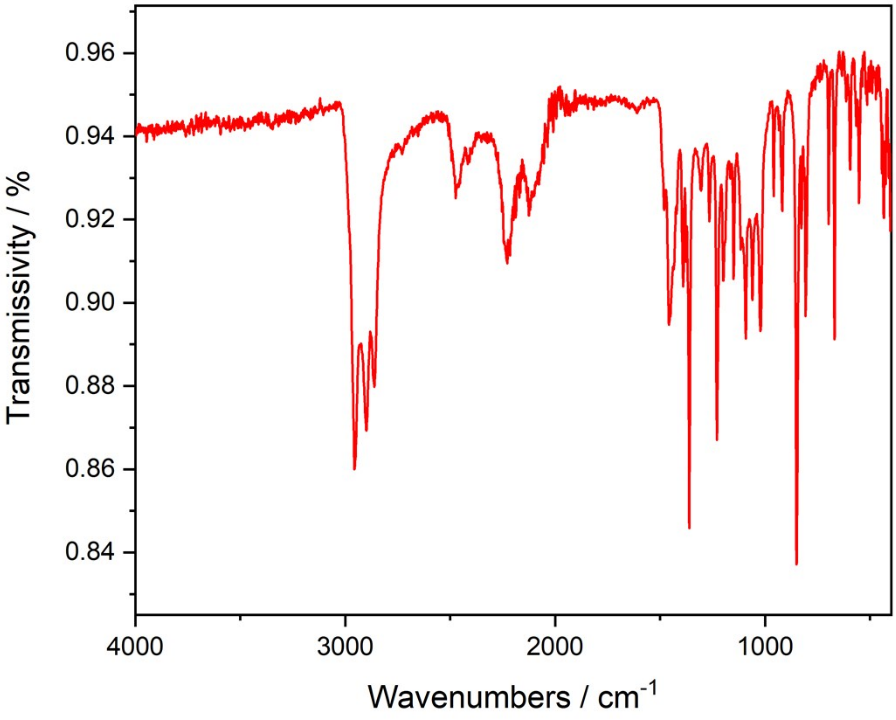

Fig. S2. Infrared spectrum of compound **2**.

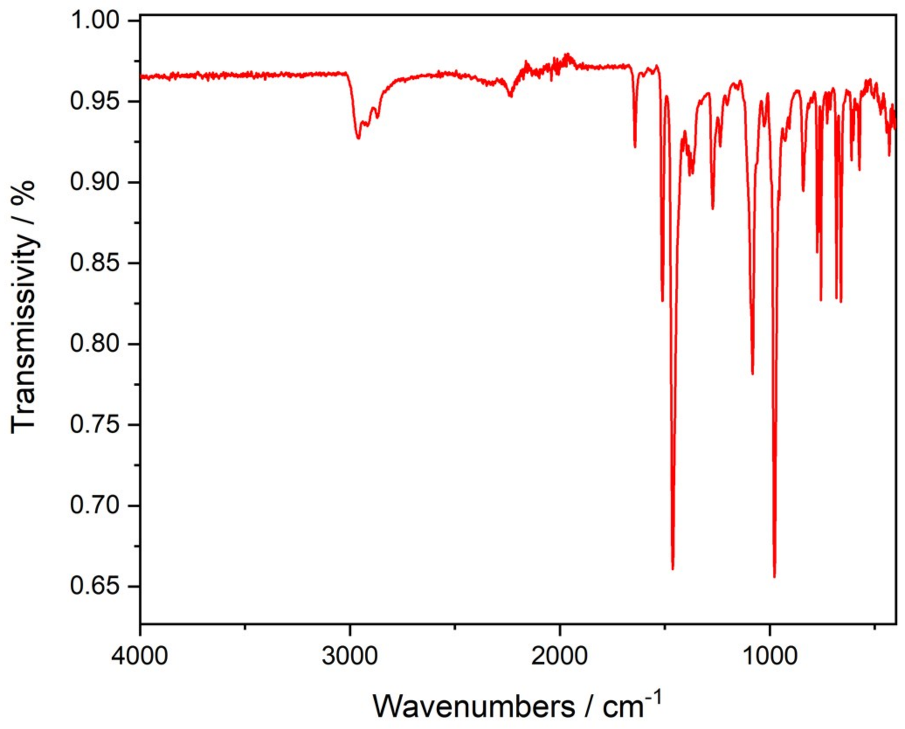

Fig. S3. Infrared spectrum of compound **3**.

X-ray crystallography

Single-crystal X-ray diffraction measurements were carried out on an Agilent Gemini Ultra diffractometer with an Enhance Ultra (Cu $K\alpha$), equipped with an Eos CCD area detector, operating in ω scanning mode to fill the Ewald sphere. Control, integration and absorption corrections were processed with the CrysAlis^{Pro} software. Crystals were mounted on MiTiGen loops from dried vacuum oil that had been kept over 4 Å molecular sieves in a glovebox under argon. Data were solved in Olex2 with SHELXT, using intrinsic phasing, and were refined with SHELXL using least squares minimisation.[4–6]

Table S1. Crystal data and structure refinement for **1-3**.

**Compound reference**	**1**	**2**	**3**
CCDC ref. code	1993006	1993008	1993009
empirical formula	$C_{34}H_{72}B_4O_2Dy_2$	$C_{46}H_{78}B_2Dy_2$	$C_{70}H_{74}B_2Dy_2F_{20}$
formula weight	881.15	977.70	1641.91
crystal system	triclinic	monoclinic	monoclinic
space group	P–1	$P2_1/n$	$P2_1/n$
a/Å	10.0067(5)	13.3199(1)	18.1029(5)
b/Å	14.2962(7)	18.1439(1)	20.9327(4)
c/Å	16.3689(8)	19.6411(2)	19.4229(5)
α/°	99.987(4)	90	90
β/°	105.704(4)	107.366(1)	111.812(3)
γ/°	107.788(4)	90	90
Volume/Å^3	2061.03(19)	4530.39(7)	6833.2(3)
Z	2	4	4
Temperature/K	220	100	100
ρ_{calc} g/cm^3	1.420	1.433	1.596
F(000)	888.0	1984.0	3264.0
Reflections collected	13654	31291	55944
Independent reflections	7793	8690	12172
R_{int}	0.0314	0.0402	0.0739
Goodness of fit on F^2	1.038	1.024	1.055
$R_1{}^a$	0.0346	0.0266	0.0692
$R_w{}^b$	0.0865	0.0655	0.1762

$^a R_1[I>2\sigma(I)] = \sum||F_o|-|F_c||/\sum|F_o|$; $^b R_w$[all data] $= [\sum\{w(F_o{}^2-F_c{}^2)^2\}/\sum\{w(F_o{}^2)^2\}]^{1/2}$

Table S2. Selected bond lengths (Å) for **1**.

	**1**
M–C	Dy1–C1: 2.698(4)<br>Dy1–C2: 2.668(4)<br>Dy1–C3: 2.607(4)<br>Dy1–C4: 2.640(4)<br>Dy1–C5: 2.641(4)<br>Dy2–C6: 2.696(4)<br>Dy2–C7: 2.668(4)<br>Dy2–C8: 2.619(4)<br>Dy2–C9: 2.648(4)<br>Dy2–C10: 2.635(4)
M–fulvalene centroid	Dy1-centroid: 2.361(1)<br>Dy2-centroid: 2.362(1)
M···M	Dy1···Dy2: 5.443(1)
M–O	Dy1–O1: 2.338(3)<br>Dy2–O2: 2.342(3)
M···B	Dy1···B1: 2.506(6)<br>Dy1···B2: 2.490(6)<br>Dy2···B3: 2.498(6)<br>Dy2···B4: 2.496(6)

Table S3. Selected bond lengths (Å) and angles (°) for **2**.

	**2**
M–C (fulvalene)	Dy1–C1: 2.653(2)<br>Dy1–C2: 2.687(3)<br>Dy1–C3: 2.670(3)<br>Dy1–C4: 2.704(3)<br>Dy1–C5: 2.630(2)<br>Dy2–C6: 2.649(3)<br>Dy2–C7: 2.677(3)<br>Dy2–C8: 2.671(3)<br>Dy2–C9: 2.698(3)<br>Dy2–C10: 2.633(3)
M–C (Cp*)	Dy1–C27: 2.643(3)<br>Dy1–C28: 2.645(3)<br>Dy1–C29: 2.659(2)<br>Dy1–C30: 2.646(2)<br>Dy1–C31: 2.667(2)<br>Dy2–C37: 2.663(3)<br>Dy2–C38: 2.660(3)<br>Dy2–C39: 2.650(3)<br>Dy2–C40: 2.630(3)<br>Dy2–C41: 2.647(3)
M–fulvalene centroid	Dy1-centroid: 2.378(1)<br>Dy2-centroid: 2.375(1)
M–Cp* centroid	Dy1-centroid: 2.362(1)<br>Dy2-centroid: 2.360(1)
M···M	Dy1···Dy2: 4.148(1)
M···B	Dy1···B1: 2.909(3)<br>Dy1···B2: 3.037(4)<br>Dy2···B1: 2.782(6)<br>Dy2···B2: 3.323(1)
centroid-M-centroid	Dy1: 137.913(1)<br>Dy2: 139.143(1)

Table S4. Selected bond lengths (Å) and angles (°) for **3**.[a]

	**3 (disordered part 1)**	**3 (disordered part 2)**
M–C (fulvalene)	Dy1–C1: 2.675(7)<br>Dy1–C2: 2.742(8)<br>Dy1–C3: 2.644(8)<br>Dy1–C4: 2.592(8)<br>Dy1–C5: 2.578(7)<br>Dy2–C6: 2.687(7)<br>Dy2–C7: 2.699(10)<br>Dy2–C8: 2.613(7)<br>Dy2–C9: 2.589(7)<br>Dy2–C10: 2.591(8)	
M–C (Cp*)	Dy1–C27: 2.634(8)<br>Dy1–C28: 2.594(7)<br>Dy1–C29: 2.599(7)<br>Dy1–C30: 2.622(8)<br>Dy1–C31: 2.650(8)<br>Dy2–C37: 2.600(16)<br>Dy2–C38: 2.620(20)<br>Dy2–C39: 2.594(18)<br>Dy2–C40: 2.599(15)<br>Dy2–C41: 2.610(15)	Dy2–C37A: 2.620(20)<br>Dy2–C38A: 2.620(20)<br>Dy2–C39A: 2.655(18)<br>Dy2–C40A: 2.699(6)<br>Dy2–C41A: 2.652 (18)
M–centroid (fulvalene)	Dy1-centroid: 2.355(1)<br>Dy2-centroid: 2.348(1)	
M–centroid (Cp*)	Dy1-centroid: 2.322(1)<br>Dy2-centroid: 2.310(1)	Dy2-centroid: 2.356(1)
Dy···Dy	Dy1–Dy2: 4.701(1)	
Dy···B	Dy1···B1: 2.715(10)<br>Dy2···B1: 2.696(5)	
centroid-M-centroid	Dy1: 145.727(1)<br>Dy2: 146.246(1)	Dy2: 145.656(1)

[a] The Cp* ligand containing C37-C41 is disordered over two sites.

Magnetic property measurements

Magnetic susceptibility measurements were recorded on a Quantum Design MPMS-XL7 SQUID magnetometer equipped with a 7 T magnet. The samples were restrained in eicosane and sealed in 7 mm NMR tubes. Direct current (DC) magnetic susceptibility measurements were performed on crystalline samples in the temperature range 1.9-300 K using an applied field of 1000 Oe. The AC susceptibility measurements were performed in zero DC field. Diamagnetic corrections were made with Pascal's constants for all the constituent atoms.[7]

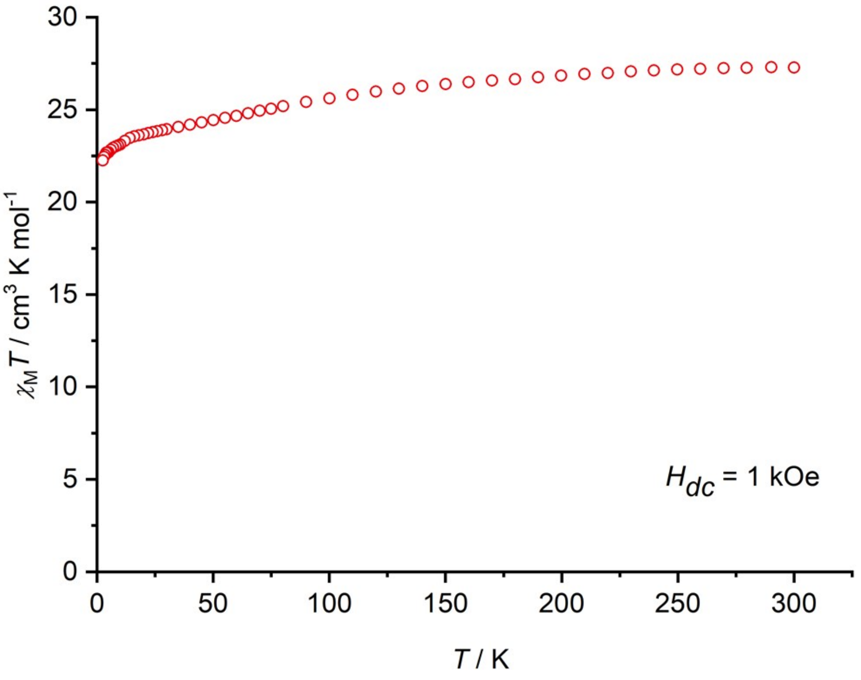

Fig. S4. Plot of $\chi_M T$ versus temperature for **1** in an applied field of 1 kOe. $\chi_M T$ (300 K) = 27.3 cm^3 K mol^{-1}, $\chi_M T$ (2.5 K) = 22.2 cm^3 K mol^{-1}.

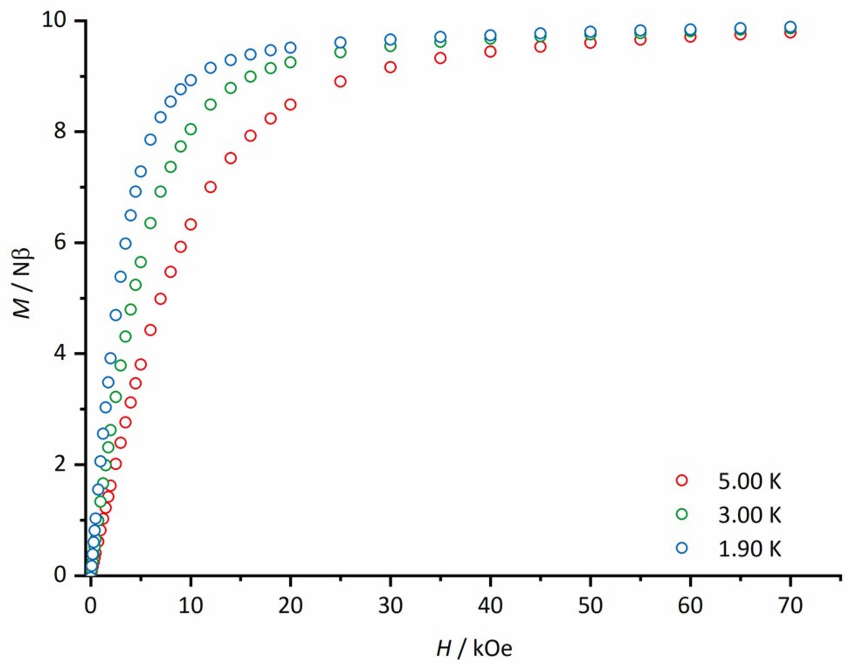

Fig. S5. Field-dependent isothermal magnetization for **1** at 1.9 K, 3.0 K and 5.0 K. The value of *M* at 1.9 K and 7 T is 9.89 Nβ.

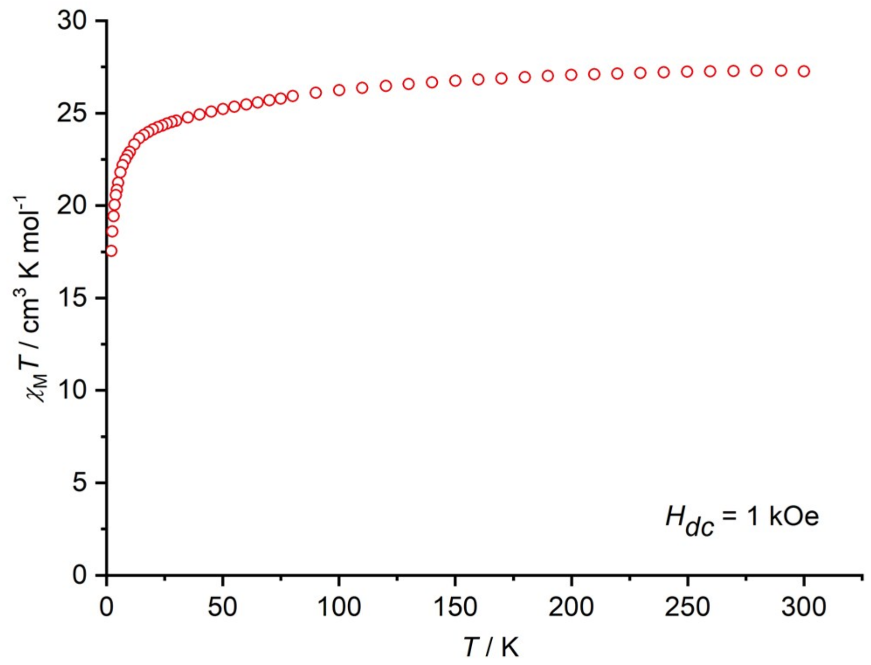

Fig. S6. Plot of $\chi_M T$ versus temperature for **2** in an applied magnetic field of 1 kOe. $\chi_M T$ (300 K) = 27.2 cm^3 K mol^{-1}, $\chi_M T$ (2.0 K) = 17.5 cm^3 K mol^{-1}.

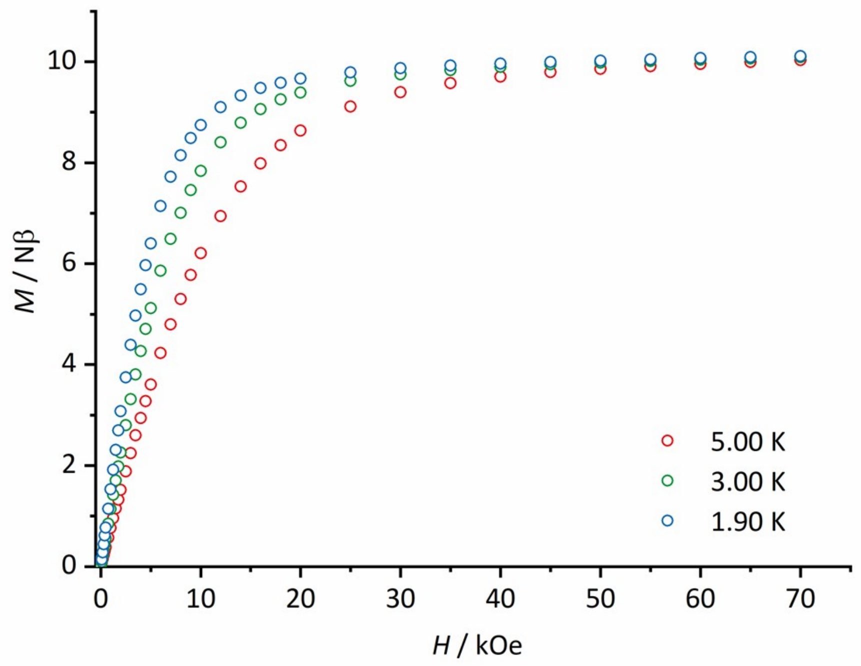

Fig. S7. Field-dependent isothermal magnetization for **2** at 1.9 K, 3.0 K and 5.0 K. The value of *M* at 1.9 K and 7 T is 10.11 Nβ.

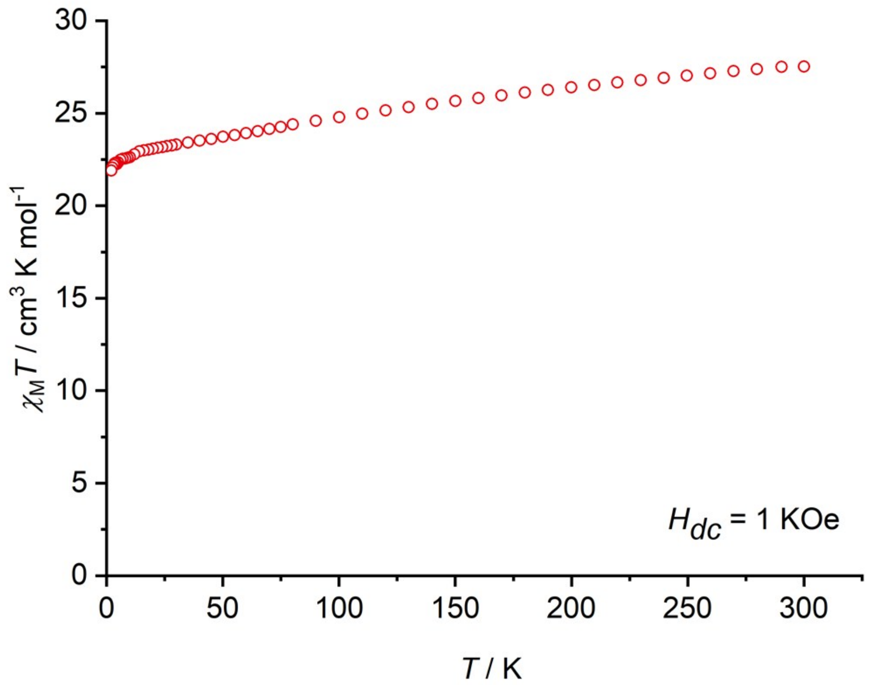

Fig. S8. Plot of $\chi_M T$ versus temperature for **3** in an applied magnetic field of 1 kOe. $\chi_M T$ (300 K) = 27.5 cm^3 K mol^{-1}, $\chi_M T$ (2.0 K) = 21.9 cm^3 K mol^{-1}.

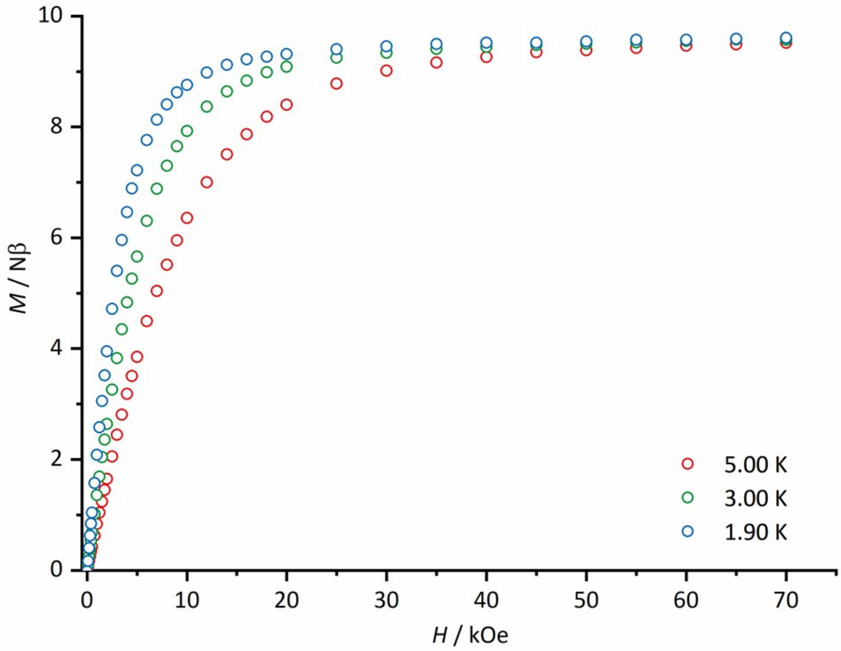

Fig. S9. Field-dependent isothermal magnetization for **3** at at 1.9 K, 3.0 K and 5.0 K. The value of *M* at 1.9 K and 7 T is 9.60 Nβ.

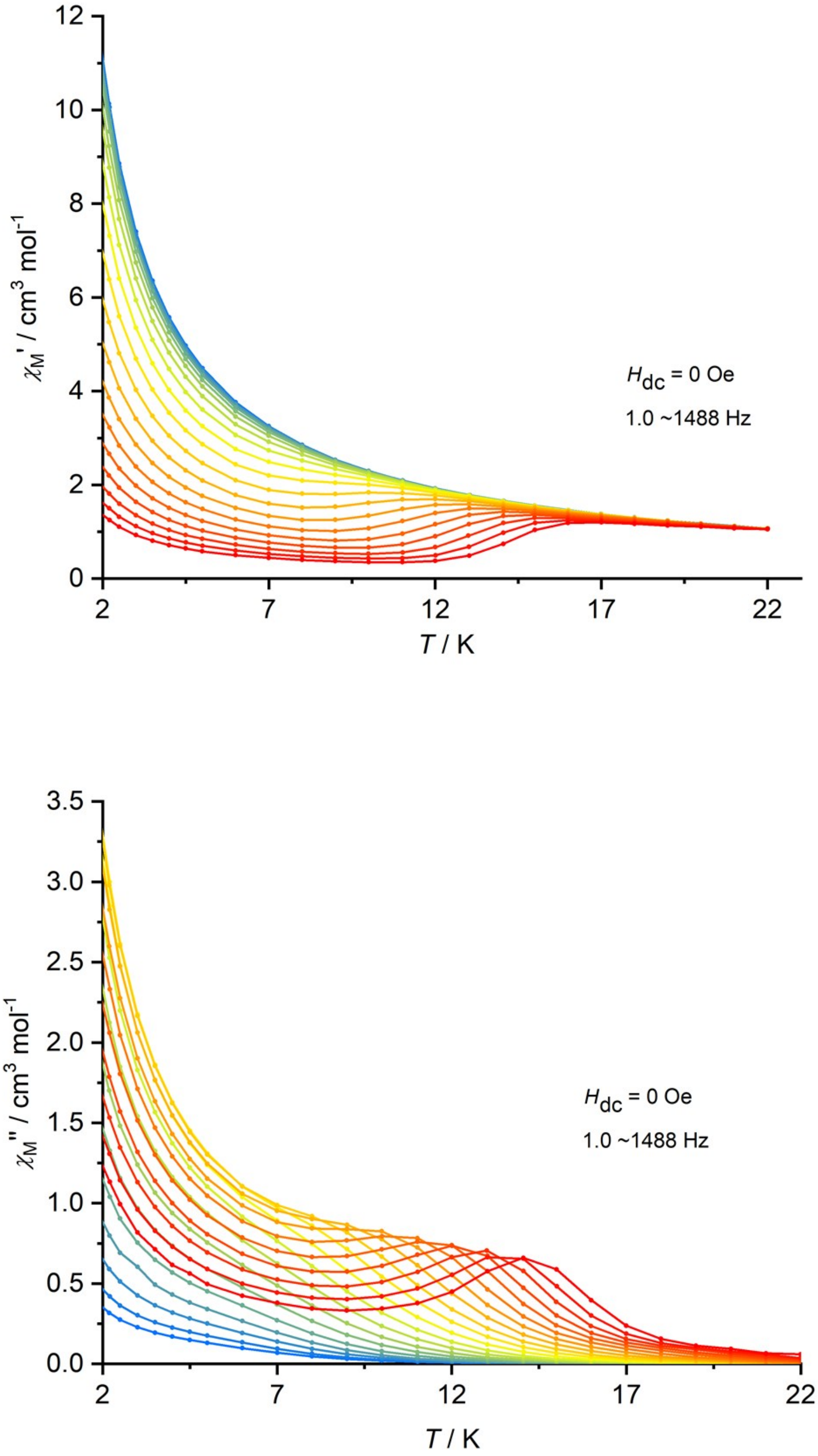

Fig. S10. Temperature dependence of the in-phase (χ'_M) and out-of-phase (χ''_M) AC susceptibility for **1** at various frequencies in the range 1 Hz (blue) to 1488 Hz (red) under zero DC field. Solid lines are a guide to the eye.

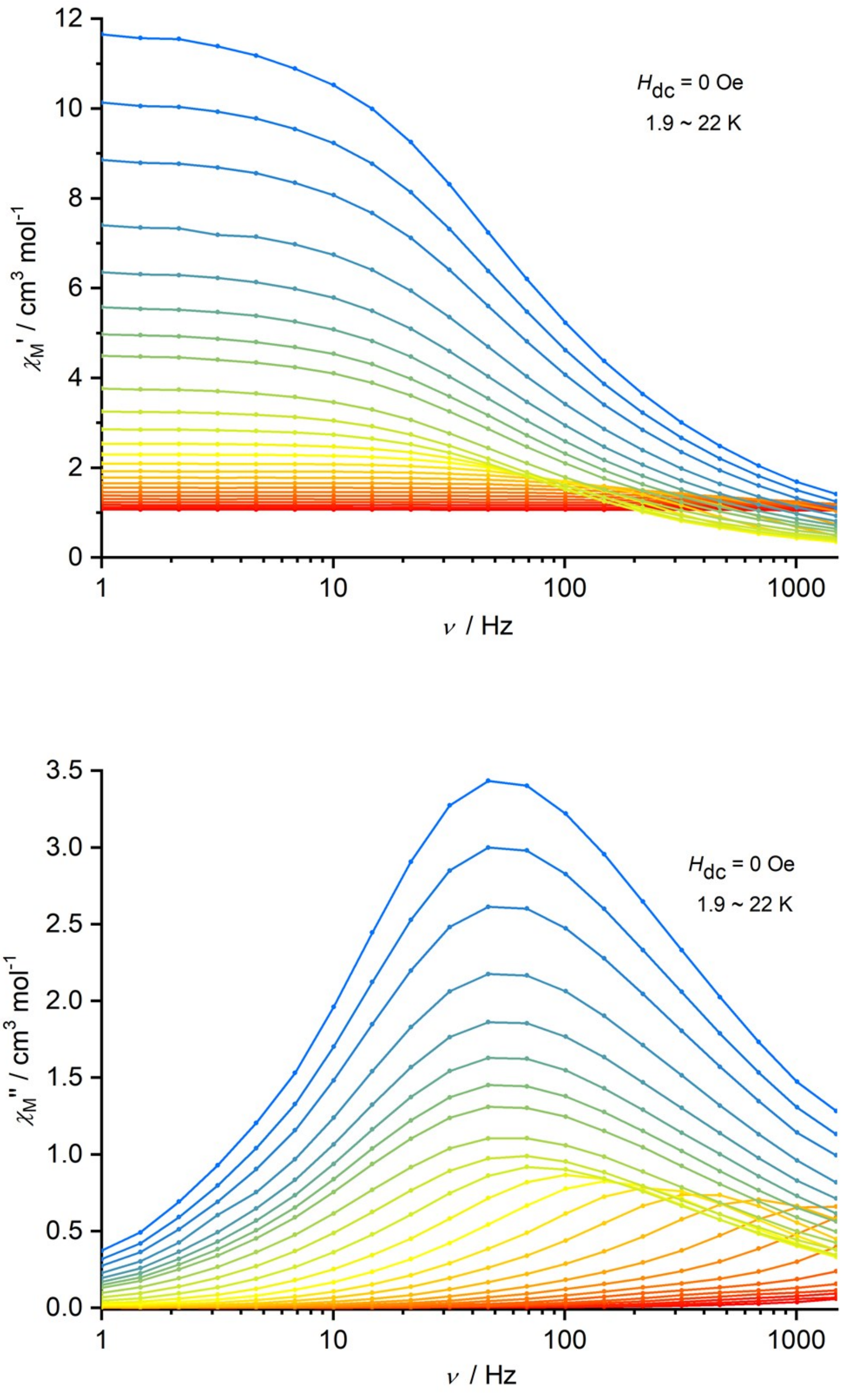

Fig. S11. Frequency dependence of the in-phase (χ'_M) and out-of-phase (χ''_M) susceptibility for **1** in zero DC field at various temperatures in the range 1.9 K (blue) to 22 K (red). Solid lines are a guide to the eye.

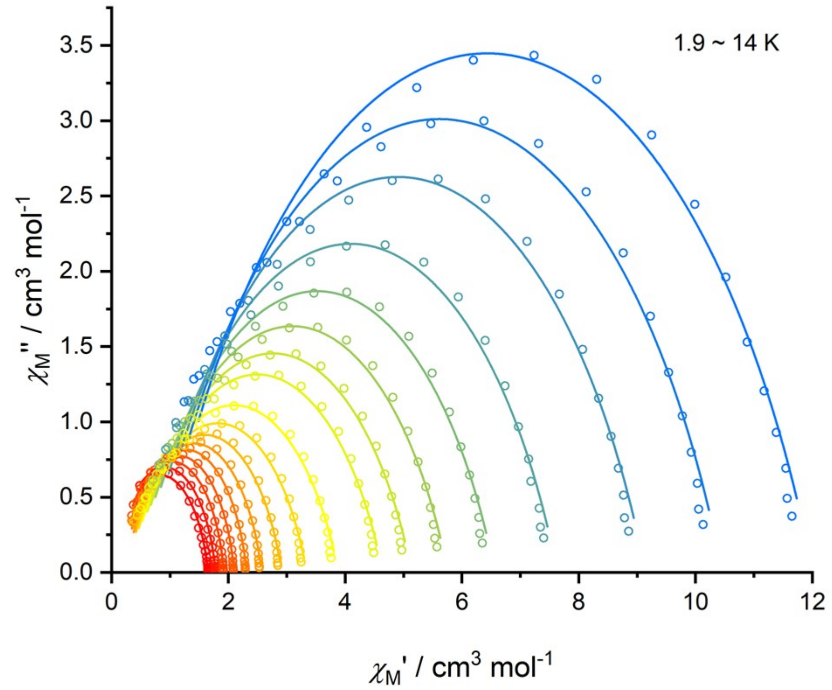

Fig. S12. Cole-Cole plots for the AC susceptibilities in zero DC field for **1** from 1.9-14 K. Solid lines represent fits to the data using equations 1 and 2, which describe χ' and χ'' in terms of frequency, isothermal susceptibility (χ_T), adiabatic susceptibility (χ_S), relaxation time (τ), and a variable representing the distribution of relaxation times (α).[7]

$$\chi'(\nu_{ac}) = \chi_\infty + \frac{(\chi_s - \chi_\infty)[1+(2\pi\nu_{ac}\tau)^{1-\alpha}\sin(\alpha\pi/2)]}{1+2(2\pi\nu_{ac}\tau)^{1-\alpha}\sin(\alpha\pi/2)+(2\pi\nu_{ac}\tau)^{2(1-\alpha)}}$$ Equation S1

$$\chi''(\nu_{ac}) = \frac{(\chi_s - \chi_\infty)(2\pi\nu_{ac}\tau)^{1-\alpha}\cos(\alpha\pi/2)}{1+2(2\pi\nu_{ac}\tau)^{1-\alpha}\sin(\alpha\pi/2)+(2\pi\nu_{ac}\tau)^{2(1-\alpha)}}$$ Equation S2

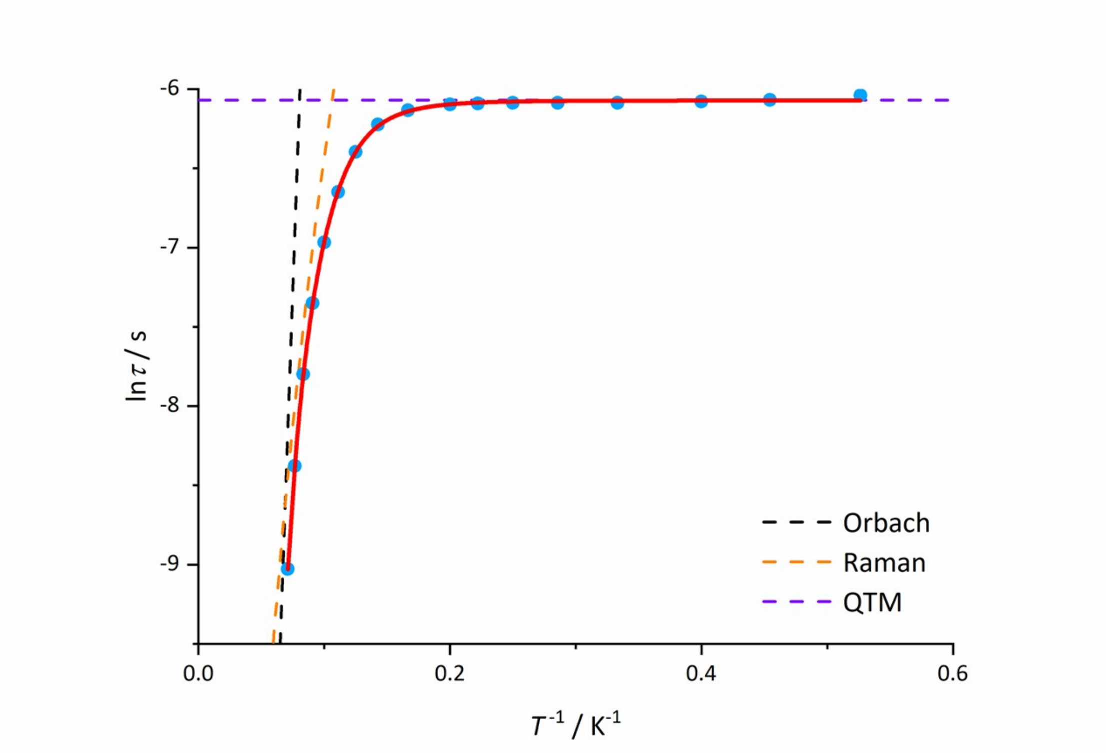

Fig. S13. Plot of natural log of the relaxation time (τ) *vs.* inverse temperature for **1**. The solid red line is the best fit (adjusted $R^2 = 0.99987$) to the equation $\tau^{-1} = \tau_0^{-1} e^{-U_{eff}/k_B T} + CT^n + \tau_{QTM}^{-1}$, giving: $U_{eff} = 154(15)$ cm^{-1}, $\tau_0 = 3.93(6) \times 10^{-11}$ s, $C = 8.16(3) \times 10^{-4}$ s^{-1} K^{-n}, $n = 5.87(1)$ and $\tau_{QTM} = 2.31(1) \times 10^{-3}$ s.

Table S5. Relaxation fitting parameters for **1** corresponding to Figures S23-S24.

T / K	χ_T / cm^3 mol^{-1}	χ_S / cm^3 mol^{-1}	α	τ / s
14.06	1.65224(0.0031)	0§	0.15263(0.00936)	1.20081E-4 (4.21E-6)
13.03	1.77967(0.00236)	0.05386(0.01303)	0.13526(0.00492)	2.29938E-4 (2.93E-6)
12	1.92129(0.00183)	0.14236(0.00619)	0.11958(0.00295)	4.10316E-4 (2.52E-6)
11	2.09779(0.00288)	0.19103(0.00734)	0.13083(0.00379)	6.429E-4 (4.84E-6)
10	2.31333(0.00579)	0.22883(0.01201)	0.15463(0.00626)	9.43192E-4 (1.19E-5)
9	2.57102(0.0095)	0.25952(0.01696)	0.18765(0.00848)	0.00129(2.30E-5)
8	2.91312(0.01406)	0.28278(0.02237)	0.22664(0.01016)	0.00167(3.77E-5)
7	3.34096(0.01838)	0.29652(0.02709)	0.26529(0.01071)	0.00198(5.04E-5)
6	3.88481(0.02151)	0.31078(0.03045)	0.29171(0.01019)	0.00217(5.49E-5)
5	4.64557(0.02498)	0.35246(0.03472)	0.30024(0.00968)	0.00225(5.49E-5)
4.5	5.14155(0.0277)	0.38302(0.03841)	0.30116(0.00966)	0.00226(5.5E-5)
4	5.76087(0.03025)	0.43(0.04184)	0.29925(0.00942)	0.00227(5.38E-5)
3.5	6.56279(0.03459)	0.48492(0.04784)	0.2982(0.00946)	0.00227(5.39E-5)
3	7.63392(0.03954)	0.56247(0.0546)	0.29588(0.00931)	0.00227(5.29E-5)
2.5	9.14251(0.04813)	0.67345(0.06613)	0.29315(0.00947)	0.00229(5.39E-5)
2.2	10.457(0.05544)	0.76801(0.07574)	0.29196(0.00952)	0.00231(5.46E-5)
1.9	12.00758(0.06184)	0.86028(0.08327)	0.29476(0.00914)	0.00238(5.41E-5)

§ These parameter values were restricted to being non-negative.

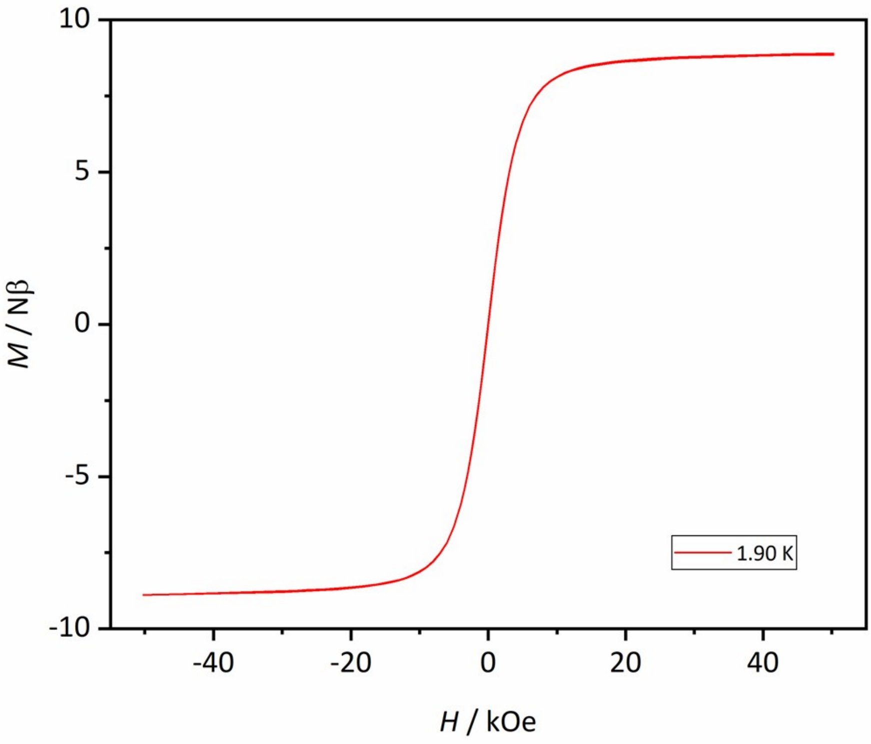

Fig. S14. Magnetic hysteresis loops for **1**. The data were collected at 1.9 K using an average field sweep rate of 23 Oe s^{-1}.

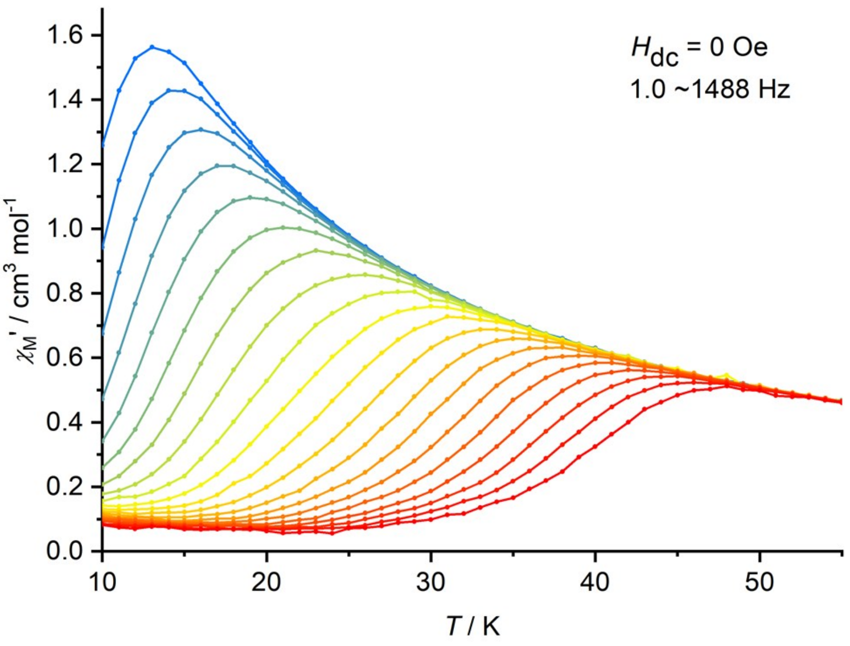

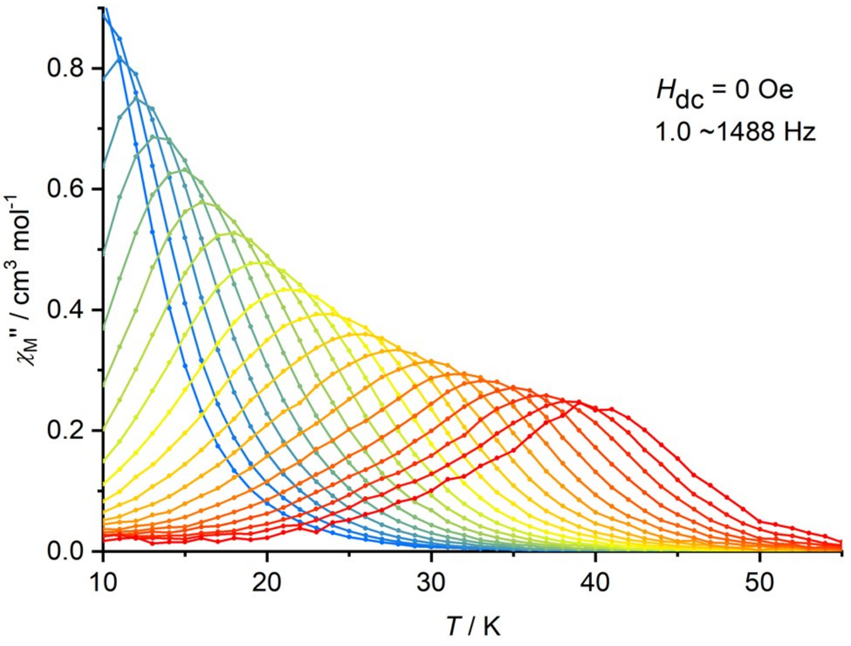

Fig. S15. Temperature dependence of the in-phase (χ'_M) and out-of-phase (χ''_M) AC susceptibility for **2** at various frequencies in the range 1 Hz (blue) to 1488 Hz (red) under zero DC field. Solid lines are a guide to the eye.

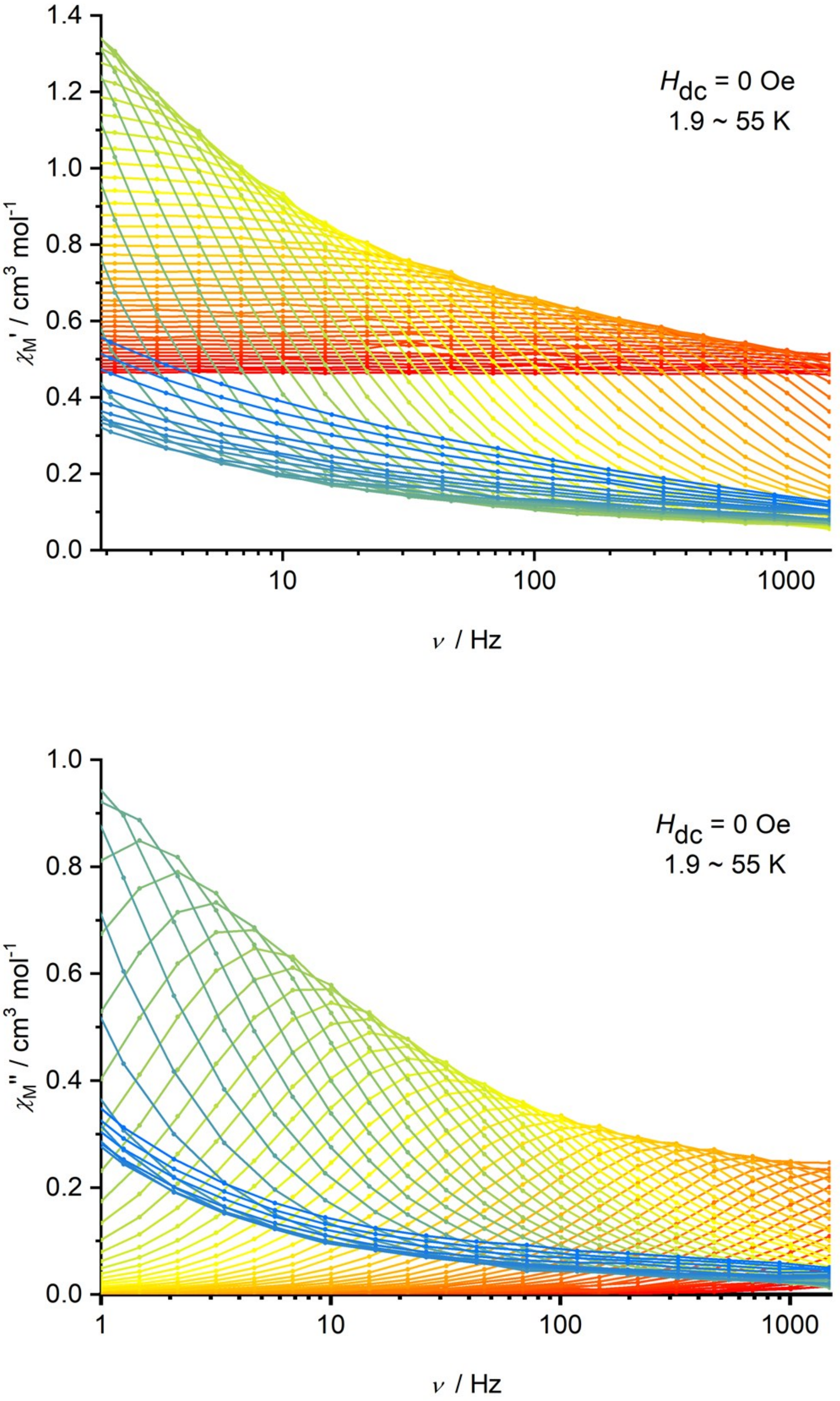

Fig. S16. Frequency dependence of the in-phase (χ'_M) and out-of-phase (χ''_M) susceptibility for **2** in zero DC field at various temperatures in the range 1.9 K (blue) to 55 K (red). Solid lines are a guide to the eye.

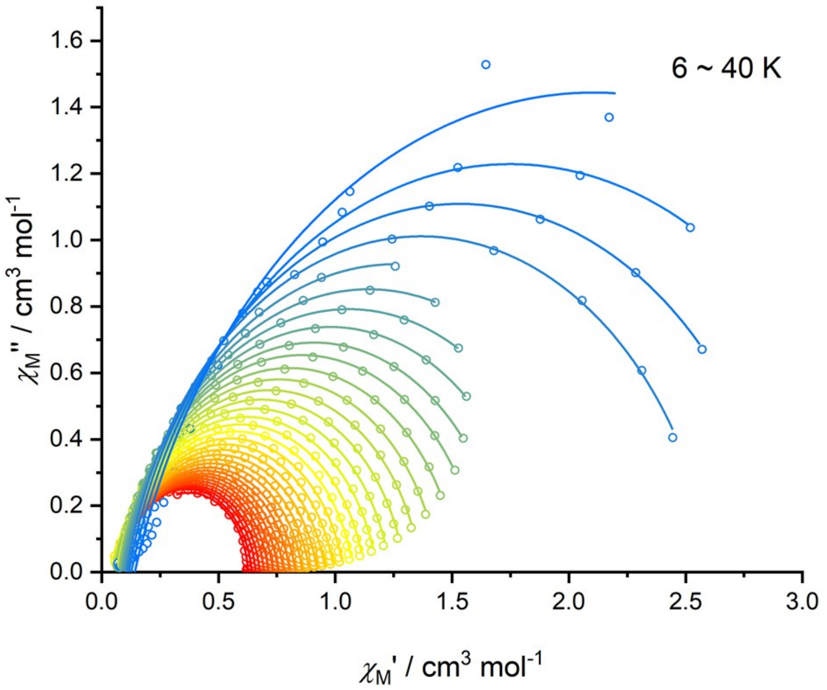

Fig. S17. Cole-Cole plots for the AC susceptibilities in zero DC field for **2** from 6-40 K. Solid lines represent fits to the data using equations S1 and S2.

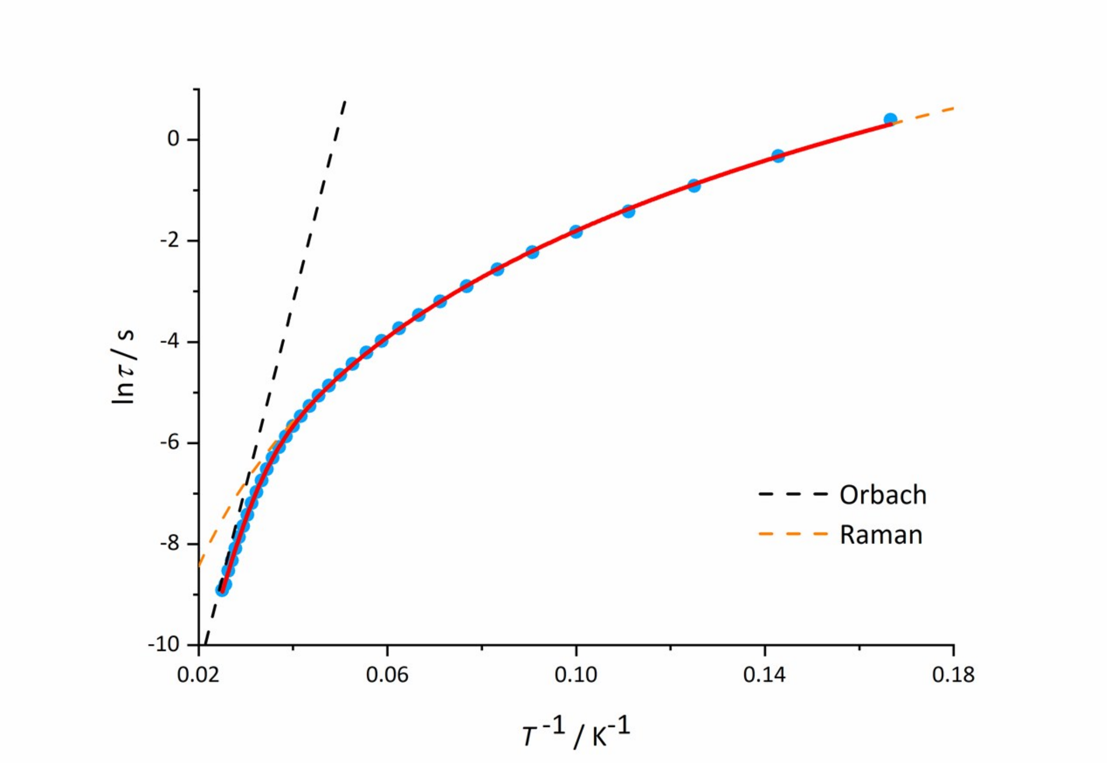

Fig. S18. Plot of natural log of the relaxation time (τ) *vs.* inverse temperature for **2**. The solid red line is the best fit (adjusted $R^2 = 0.99991$) to equation $\tau^{-1} = \tau_0^{-1} e^{-U_{eff}/k_B T} + CT^n$, giving: $U_{\text{eff}} = 252(4)$ cm^{-1}, $\tau_0 = 1.94(3) \times 10^{-8}$ s, $C = 4.56\ (2) \times 10^{-4}$ s^{-1} K^{-n}, $n = 4.12(1)$.

Table S6. Relaxation fitting parameters for **2** corresponding to Figures S33-S34.

T / K	χ_T / cm^3 mol^{-1}	χ_S / cm^3 mol^{-1}	α	τ / s
40	0.62631(5.08E-4)	0.11981(0.00451)	0.03639(0.00464)	1.34283E-4(1.62E-6)
39	0.64266(8.14E-4)	0.08219(0.00647)	0.06929(0.00634)	1.51005E-4(2.53E-6)
38	0.65831(6.64E-4)	0.09873(0.00392)	0.05898(0.0045)	1.97059E-4(1.99E-6)
37	0.67575(8.98E-4)	0.09391(0.0044)	0.0698(0.00534)	2.42631E-4(2.75E-6)
36	0.69388(9.51E-4)	0.09755(0.00381)	0.0701(0.00501)	3.06877E-4(3.04E-6)
35	0.71298(8.88E-4)	0.09445(0.00302)	0.07609(0.00418)	3.83478E-4(3.05E-6)
34	0.73338(0.00136)	0.09147(0.00399)	0.08271(0.00576)	4.78412E-4(5.13E-6)
33	0.7554(0.00158)	0.08733(0.00406)	0.09098(0.00602)	5.9663E-4(6.63E-6)
32	0.77858(0.00189)	0.08599(0.00426)	0.09871(0.00653)	7.51126E-4(9.02E-6)
31	0.80468(0.00254)	0.08398(0.00508)	0.10429(0.00796)	9.39476E-4(1.37E-5)
30	0.82926(0.00231)	0.07956(0.00413)	0.10946(0.00661)	0.00118(1.42E-5)
29	0.85913(0.00282)	0.079(0.00449)	0.11053(0.00732)	0.00148(1.97E-5)
28	0.88923(0.00325)	0.07754(0.00468)	0.11354(0.00774)	0.00184(2.60E-5)
27	0.92247(0.00345)	0.0742(0.0045)	0.11604(0.00749)	0.00228(3.12E-5)
26	0.95737(0.00377)	0.07072(0.00448)	0.11743(0.00748)	0.00281(3.85E-5)
25	0.99482(0.00373)	0.06845(0.00404)	0.11754(0.00677)	0.00345(4.27E-5)
24	1.0358(0.00398)	0.06575(0.00394)	0.1181(0.00659)	0.00422(5.09E-5)
23	1.0786(0.0038)	0.06623(0.00345)	0.1129(0.00578)	0.00515(5.41E-5)
22	1.12603(0.0036)	0.06626(0.00298)	0.11096(0.00499)	0.00634(5.74E-5)
21	1.1787(0.00351)	0.06444(0.00266)	0.11107(0.00442)	0.00774(6.22E-5)
20	1.23448(0.0031)	0.06694(0.00213)	0.10731(0.00355)	0.00954(6.13E-5)
19	1.29855(0.00279)	0.06826(0.00173)	0.10692(0.00287)	0.01186(6.18E-5)
18	1.36856(0.00317)	0.06973(0.00176)	0.10741(0.00291)	0.0148(7.86E-5)
17	1.44545(0.0032)	0.07178(0.00157)	0.10672(0.0026)	0.01871(8.97E-5)
16	1.53454(0.00417)	0.07419(0.00177)	0.10914(0.00295)	0.02402(1.33E-4)
15	1.63439(0.00445)	0.07604(0.00161)	0.11132(0.0027)	0.03126(1.64E-4)
14.05	1.7414(0.00497)	0.08038(0.00149)	0.11614(0.00256)	0.0408(2.15E-4)
13.02	1.86995(0.00796)	0.08475(0.00189)	0.12007(0.00336)	0.05515(4.16E-4)
12	2.02548(0.01419)	0.08829(0.0025)	0.12724(0.00471)	0.07692(9.35E-4)
11.02	2.19284(0.02382)	0.09336(0.00296)	0.13202(0.00607)	0.10824(0.00202)
10	2.41217(0.04278)	0.09985(0.00333)	0.13934(0.00775)	0.16131(0.00491)
9	2.62604(0.01423)	0.10686(0.00465)	0.1387(0.00554)	0.24186(0.00282)
8	2.94926(0.0215)	0.11607(0.00509)	0.1543(0.00619)	0.40079(0.00584)
7	3.3717(0.04325)	0.12681(0.00638)	0.17469(0.00834)	0.72298(0.01785)
6	4.07094(0.16648)	0.14183(0.01198)	0.19287 (0.01798)	1.47795(0.1151)

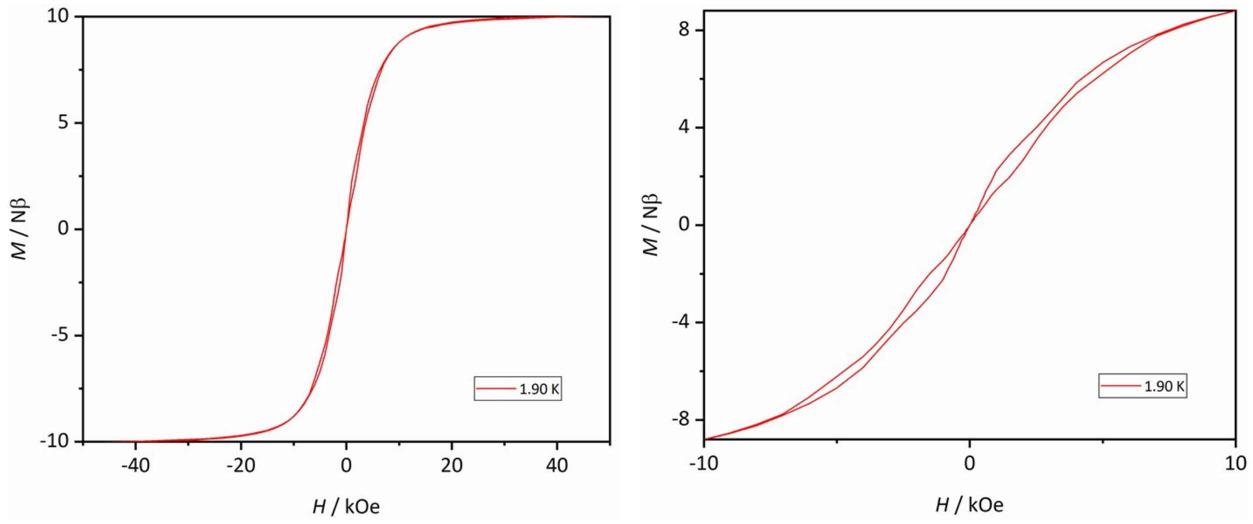

Fig. S19. Magnetic hysteresis loops for **2**. The data were collected at 1.9 K under an average field sweep rate of 23 Oe s^{-1}.

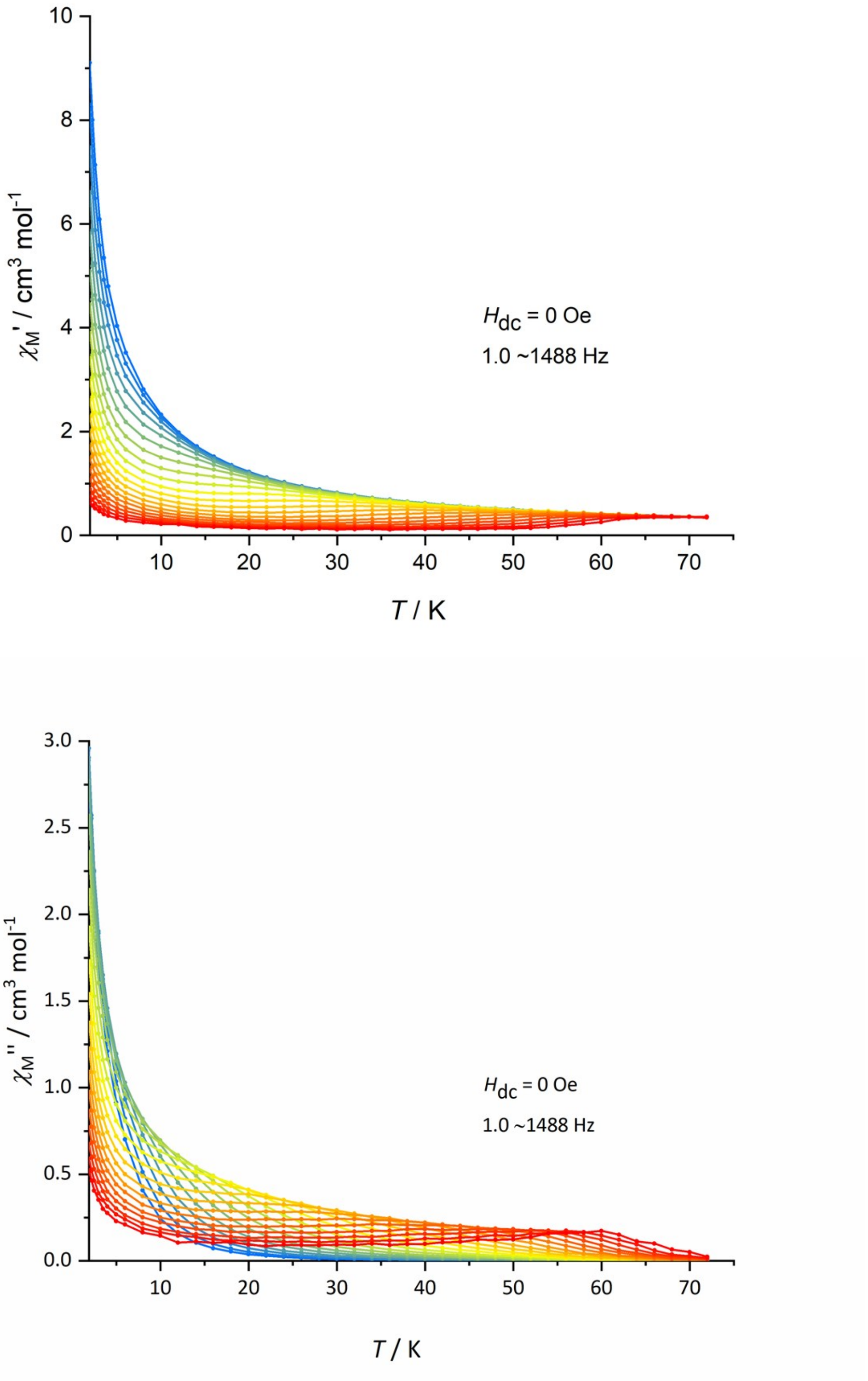

Fig. S20. Temperature dependence of the in-phase (χ'_M) and out-of-phase (χ''_M) AC susceptibility for **3** at various frequencies in the range 1 Hz (blue) to 1488 Hz (red) under zero DC field. Solid lines are a guide to the eye.

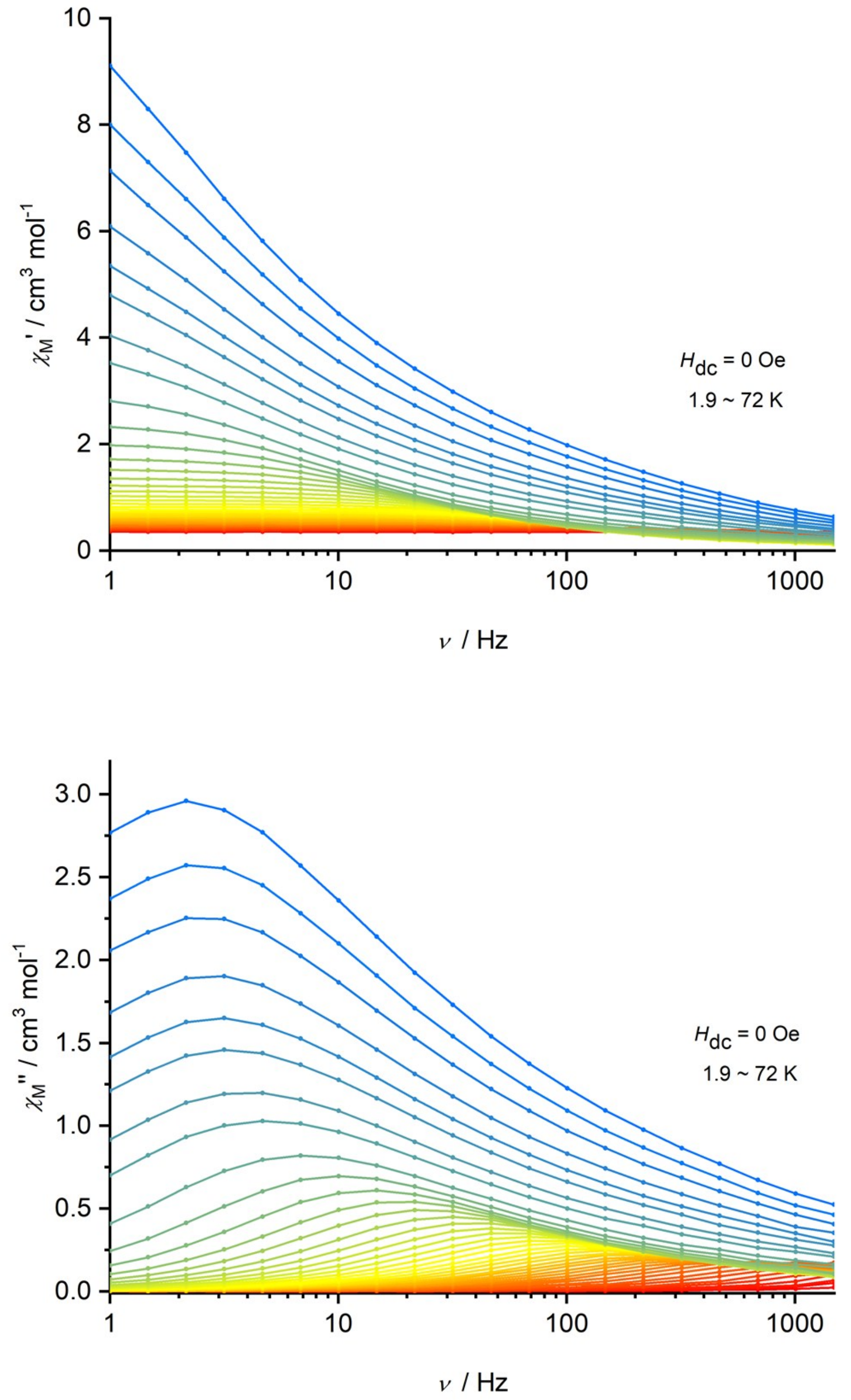

Fig. S21. Frequency dependence of the in-phase (χ'_M) and out-of-phase (χ''_M) susceptibility for **3** in zero DC field at various temperatures in the range 1.9 K (blue) to 72 K (red). Solid lines are a guide to the eye.

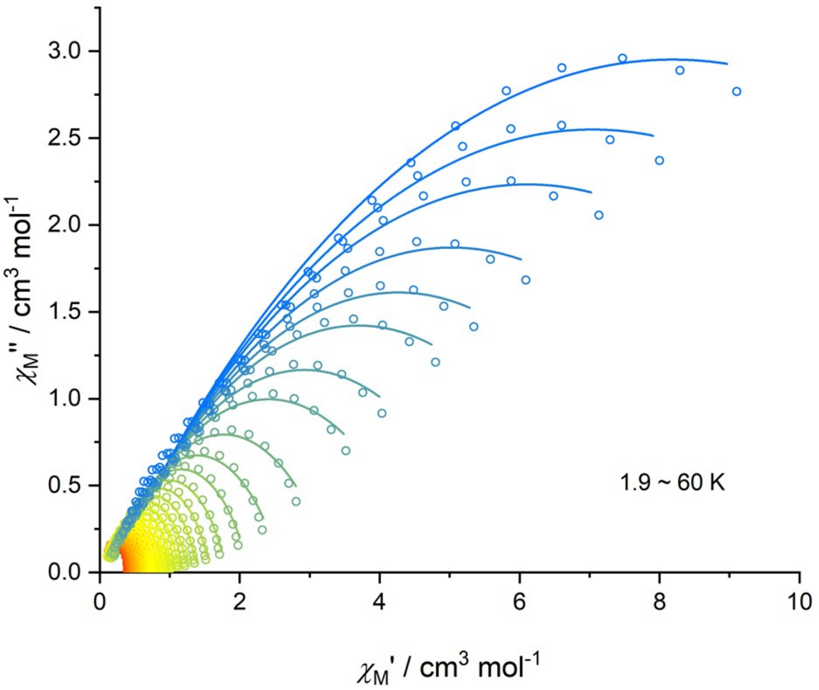

Fig. S22. Cole-Cole plots for the AC susceptibilities in zero DC field for **3** from 2-60 K. Solid lines represent fits to the data using equations S1 and S2.

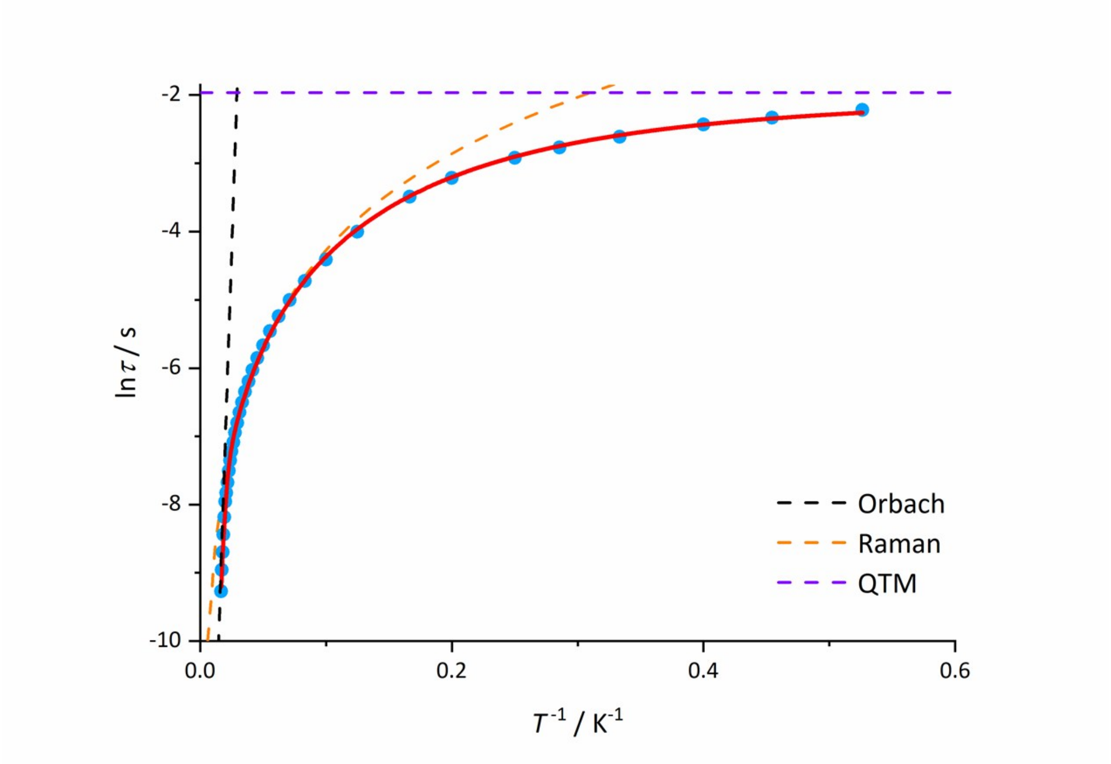

Fig. S23. Plot of natural log of the relaxation time (τ) *vs.* inverse temperature for **3**. The blue points are from the AC susceptibility measurements. The solid red line is the best fit (adjusted R^2 = 0.99963) to equation $\tau^{-1} = \tau_0^{-1} e^{-U_{eff}/k_B T} + CT^n + \tau_{QTM}^{-1}$, giving: U_{eff} = 384(18) cm^{-1}, τ_0 = 1.37(6) × 10^{-8} s, C = 6.55(4) × 10^{-1} s^{-1} K^{-n}, n = 2.03(2) and τ_{QTM} = 1.39 (6) × 10^{-1} s.

Table S7. Relaxation fitting parameters for **3** corresponding to Figures S40-S41.

T / K	χ_T / cm^3 mol^{-1}	χ_S / cm^3 mol^{-1}	α	τ / s
60	0.41817(5.29E-4)	0.0615(0.00752)	0.02535(0.00864)	9.43398E-5(2.64E-6)
58	0.43313(5.94E-4)	0.07291(0.00559)	0.05029(0.00781)	1.28887E-4(2.79E-6)
56	0.44755(4.43E-4)	0.06384(0.00314)	0.0666(0.00477)	1.67114E-4(1.98E-6)
54	0.46511(9.49E-4)	0.06115(0.00528)	0.08383(0.00861)	2.15456E-4(4.29E-6)
52	0.48126(5.38E-4)	0.06971(0.00235)	0.08024(0.00428)	2.79484E-4(2.49E-6)
50	0.50038(7.50E-4)	0.07553(0.0028)	0.09832(0.00534)	3.49799E-4(3.83E-6)
48	0.52032(0.00104)	0.0708(0.00356)	0.11221(0.00668)	3.9843E-4(5.48E-6)
46	0.5435(0.00113)	0.06923(0.00353)	0.12607(0.00657)	4.63012E-4(6.28E-6)
44	0.56682(0.00123)	0.07852(0.0034)	0.12038(0.00657)	5.50927E-4(7.18E-6)
42	0.59284(0.00125)	0.08215(0.00317)	0.12602(0.00613)	6.40314E-4(7.73E-6)
40	0.62221(0.00147)	0.0834(0.00344)	0.1258(0.00658)	7.30166E-4(9.33E-6)
38	0.65381(0.00134)	0.08515(0.00293)	0.13076(0.00551)	8.34743E-4(8.91E-6)
36	0.68994(0.00168)	0.08802(0.00339)	0.1328(0.00627)	9.63924E-4(1.16E-5)
34	0.73071(0.00224)	0.09288(0.0042)	0.1425(0.00759)	0.00111(1.64E-5)
32	0.77451(0.00228)	0.09708(0.00395)	0.14123(0.00702)	0.0013(1.75E-5)
30	0.82737(0.00303)	0.09916(0.00489)	0.14839(0.00836)	0.0015(2.43E-5)
28	0.88666(0.00313)	0.10871(0.00469)	0.1529(0.00779)	0.00175(2.66E-5)
26	0.95573(0.00402)	0.1112(0.00561)	0.16284(0.00886)	0.00204(3.57E-5)
24	1.03721(0.00476)	0.11724(0.00615)	0.17212(0.00923)	0.00241(4.45E-5)
22	1.1337(0.0058)	0.12377(0.00691)	0.18556(0.00978)	0.00287(5.72E-5)
20	1.24977(0.00755)	0.13349(0.00823)	0.19959(0.01091)	0.00346(7.85E-5)
18	1.39346(0.00972)	0.1463(0.00956)	0.21637(0.0118)	0.00426(1.07E-4)
16	1.57556(0.01244)	0.15706(0.01095)	0.23938(0.01232)	0.00531(1.45E-4)
14.05	1.80742(0.01699)	0.16857(0.01315)	0.26784(0.0133)	0.00671(2.10E-4)
12	2.13018(0.02241)	0.1897(0.01474)	0.30082(0.01319)	0.00889(2.99E-4)
10	2.59703(0.03403)	0.19585(0.01836)	0.34904(0.01381)	0.01214(4.94E-4)
8	3.33853(0.05042)	0.19888(0.02077)	0.4037(0.01263)	0.01824(8.54E-4)
6	4.63934(0.08561)	0.18721(0.02503)	0.4639(0.0115)	0.03054(0.00184)
5	5.66126(0.10864)	0.18923(0.02655)	0.48719(0.01039)	0.04024(0.00261)
4	7.21053(0.15026)	0.18399(0.0305)	0.51062(0.00973)	0.05397(0.00396)
3.5	8.34882(0.18564)	0.17699(0.03431)	0.52169(0.00963)	0.06283(0.00501)
3	9.87787(0.23036)	0.17392(0.03871)	0.53163(0.00939)	0.07345(0.00626)
2.5	12.06304(0.30031)	0.16088(0.04534)	0.54286(0.00923)	0.08787(0.00817)
2.2	13.91804(0.34953)	0.15956(0.04971)	0.54814(0.00891)	0.09708(0.0092)
1.9	16.20812(0.46213)	0.16343(0.06123)	0.55109(0.00968)	0.10851(0.01174)

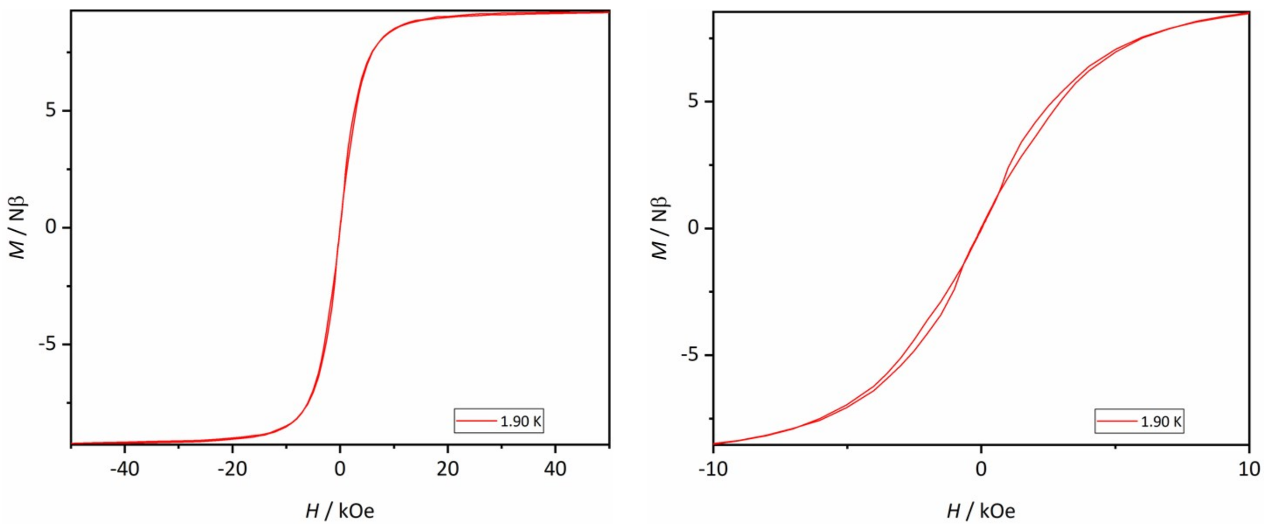

Fig. S24. Magnetic hysteresis loops for **3**. The data were collected at 1.9 K under an average field sweep rate of 23 Oe s^{-1}.

Computational Details

The geometries used in the calculations were extracted from the crystal structure. The positions of hydrogen atoms were optimized using density functional theory (DFT) while the positions of heavier atoms were kept frozen to their crystal-structure coordinates. The DFT optimization were carried out using the ADF 2019 code.[8,9] The pure PBE generalized gradient approximation to the exchange-correlation functional[10,11] was used along with Grimme's empirical DFT-D3 dispersion correction[12] with the Becke–Johson (BJ) damping function.[13] Scalar relativistic effect were taken into account using the zeroth-order regular approximation (ZORA).[14–16] Slater-type all-electron basis sets specifically designed for ZORA calculations were utilized in the optimizations.[17] A valence triple-ζ quality basis with two sets of polarization functions (TZ2P) was used for the Dy ions while polarized valence double-ζ quality (DZP) bases were used for the other atoms. In order to simulate static electron correlation effects at the Dy ions, the unpaired electrons were equally distributed over the seven 4*f* orbitals of the two Dy ions yielding fractional occupation numbers. The "NumericalQuality" keyword in ADF was set to "Good" and the geometry convergence thresholds were increased to 10^{-4}, 10^{-4}, 10^{-3} and 10^{-1} atomic units for energy, energy gradient, bond length and bond angle, respectively.

Each of the two Dy^{3+} ions in **1**, **2** and **3** were treated in a separate multireference calculations while the other ion was replaced by a diamagnetic Y^{III} ion. The multireference calculations were carried out using the *OpenMolcas* code version 19.11.[18] First a state-averaged (SA) complete active space self-consistent field (CASSCF) calculation was carried out separately for each multiplicity of each ion.[19–23] The active space consisted of the nine 4*f* electrons in the seven 4*f* orbitals. All 21 sextets, 224 quartets and 490 doublets were solved with equal weights. All 21 sextets, the lowest 128 quartets and the lowest 130 doublets (corresponding to an energy-cutoff of 50,000 cm^{-1}) were then used as a basis for the construction of the spin-orbit coupling (SOC) operator following the spin-orbit restricted active space state interaction (SO-RASSI) formalism.[24] The operator was diagonalized to yield the spin-orbit coupled eigenstates.

The local magnetic properties (**g**-tensors, ab initio CF parameters and the transition magnetic moment matrix elements) were evaluated using the SINGLE_ANISO_OPEN module[25,26] of *OpenMolcas*. The exchange interactions were calculated using the POLY_ANISO module.[26–28] The dipolar interaction was calculated using the local **g**-tensors and the point-dipole approximation. Eight lowest KDs of each Dy site were used in construction of the exchange operator. The Lines exchange parameter[29] was determined by fitting the calculated magnetic susceptibility data to experiment. The fit was carried out by scanning the exchange parameter first from –6.00 cm^{-1} to 6.00 cm^{-1} in 0.01 cm^{-1} increments. Then, in the vicinity of the the minimal standard deviations a new scan was carried out using 0.0001 cm^{-1} increments. The eigenvalues obtained by diagonalization of the Lines exchange operator and the point-dipole coupling operator where mapped to the eigenvalues of an Ising-type Hamiltonian acting on two pseudospin doublets. The Ising-type exchange parameters are identified as double the energy difference between the two lowest eigenstate doublets. Note that the two states in each doublet are not exactly degenerate, but the splittings are less than 10^{-4} cm^{-1}.

Relativistically contracted natural atomic orbital basis sets were used in all multireference calculations.[30–33] A polarized valence quadruple-ζ basis (VQZP) was used for the Dy ions; polarized valence triple-ζ basis sets (VTZP) were used for the H and C atoms in the coordinated Cp rings, the H and B atoms in the $[BH_4]^-$ anions and the C and O atoms in THF molecules; polarized double-ζ basis sets (VDZP) were used for the Y ions, the H atoms in the THF molecules and the C atoms in the Cp substituent groups; and a double-ζ basis without polarization functions (VDZ) was used for the H atoms in the Cp substituent groups. Cholesky decomposition with a threshold of 10^{-8} atomic units was used to store the two-electron integrals. Scalar relativistic effects were treated with the exact two-component (X2C) transformation[34–36] as implemented in *OpenMolcas*. The SOC operator was constructed using the atomic mean-field integral (AMFI) formalism.[37,38]

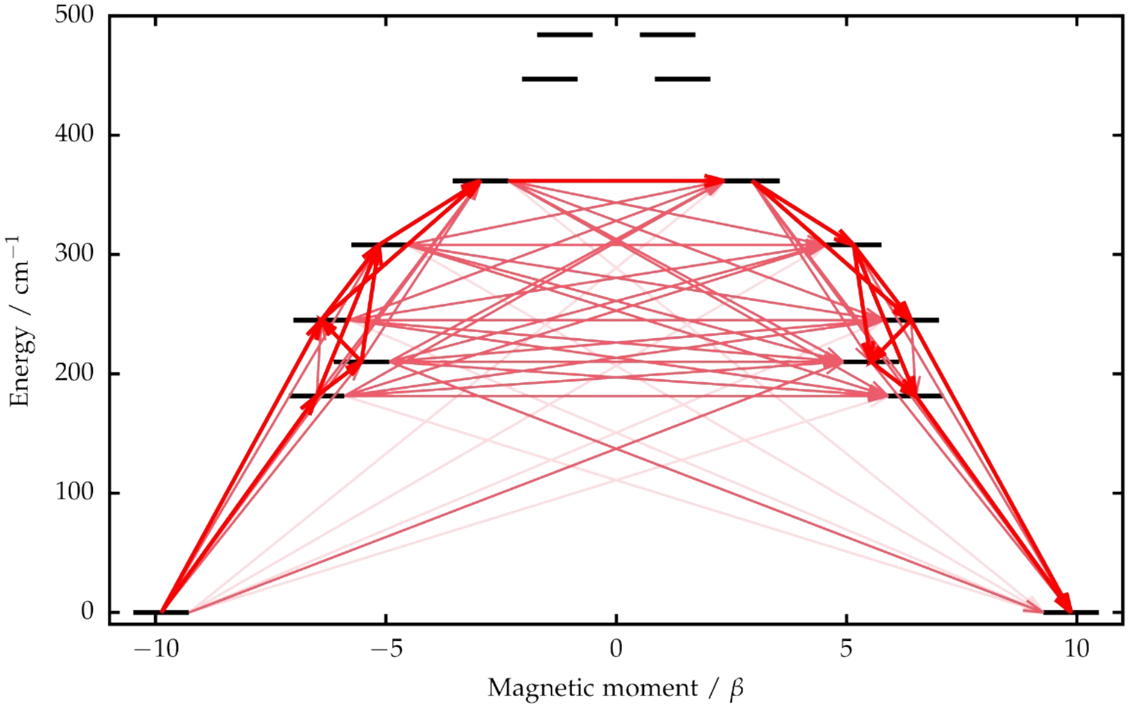

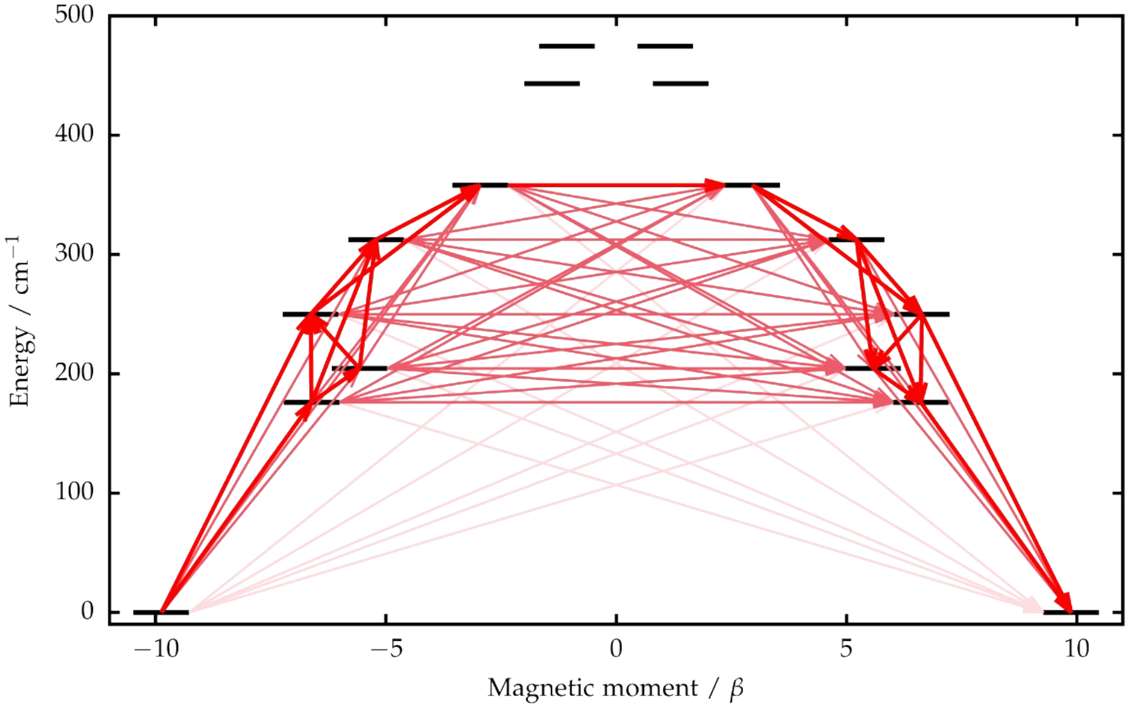

Fig. S25. Calculated effective ab initio barriers for the local relaxation of magnetization at ions Dy1 (right) and Dy2 (left) in **1**. Stronger arrows indicate larger absolute value of the transition magnetic moment matrix elements between the respective states. Transitions involving higher-energy states not involved in the relaxation mechanism are omitted for clarity.

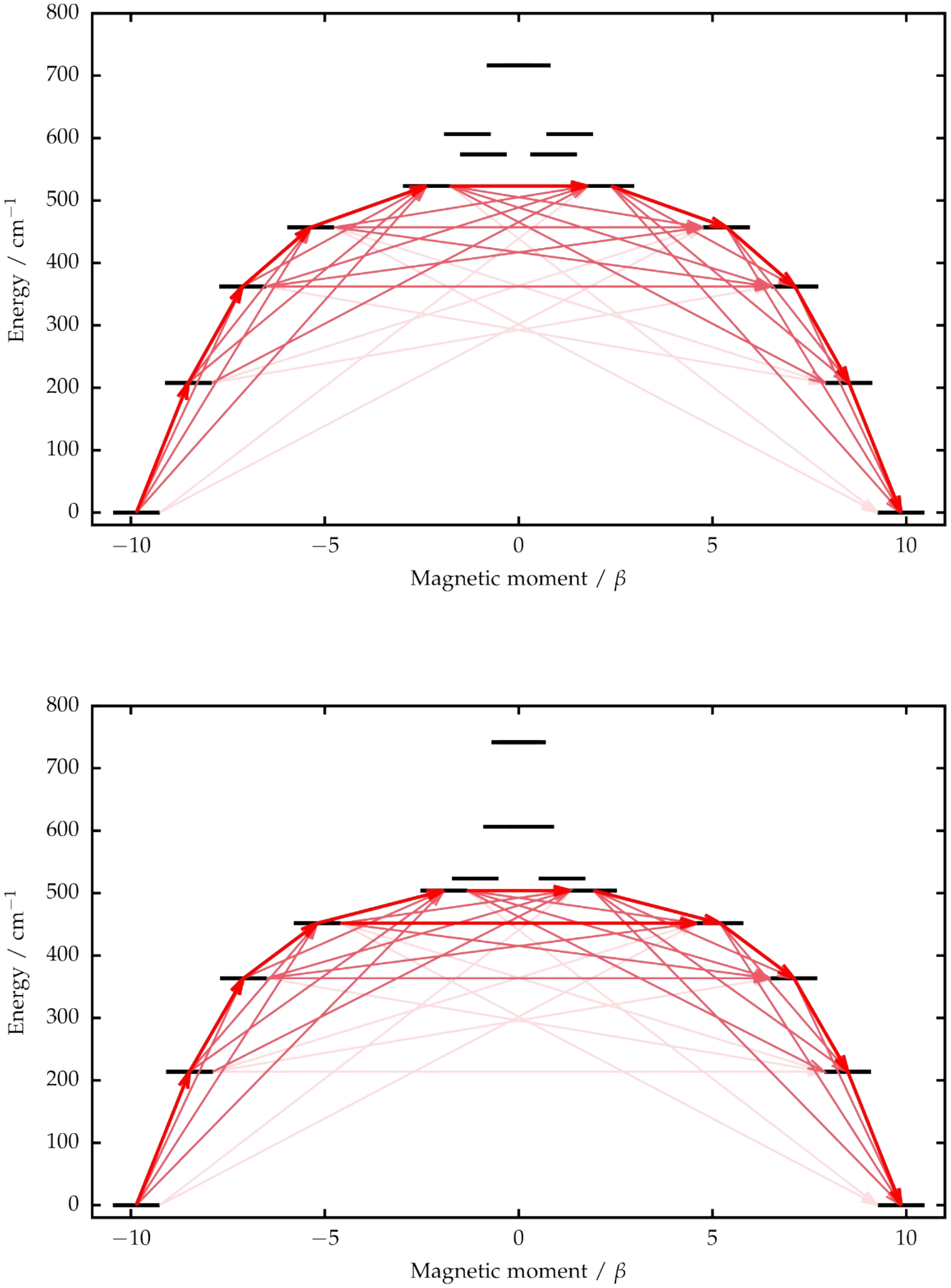

Fig. S26. Calculated effective ab initio barriers for the local relaxation of magnetization at ions Dy1 (right) and Dy2 (left) in **2**. Stronger arrows indicate larger absolute value of the transition magnetic moment matrix elements between the respective states. Transitions involving higher-energy states not involved in the relaxation mechanism are omitted for clarity.

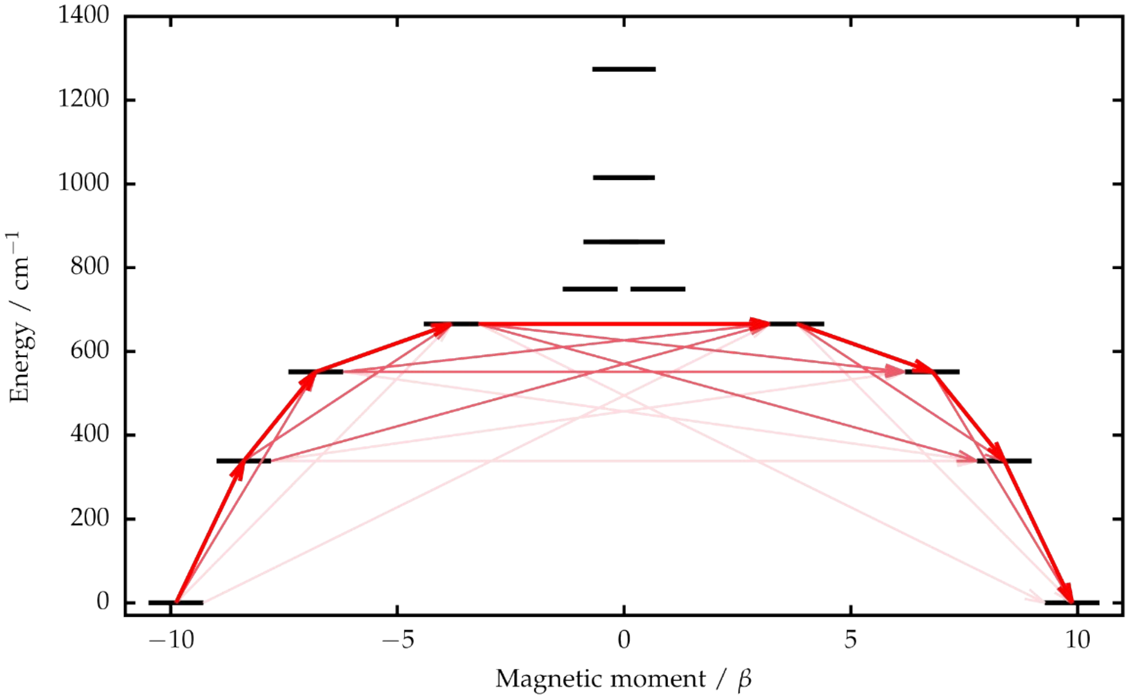

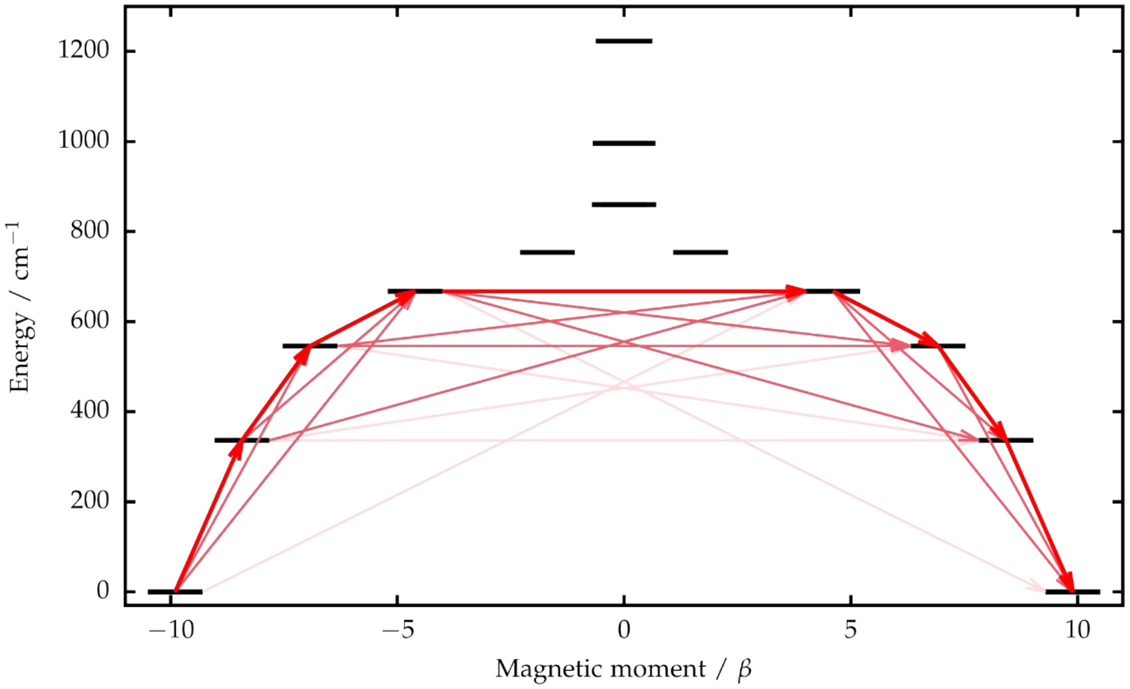

Fig. S27. Calculated effective ab initio barriers for the local relaxation of magnetization at ions Dy1 (right) and Dy2 (left) in **3**. Stronger arrows indicate larger absolute value of the transition magnetic moment matrix elements between the respective states. Transitions involving higher-energy states not involved in the relaxation mechanism are omitted for clarity.

Table S8. Properties of the eight lowest KDs of the Dy1 ion in **1** corresponding to the crystal-field split states in the ground $^6H_{15/2}$ multiplet.

KD	E / cm^{-1}	g_x	g_y	g_z	θ / ° [a]
KD1	0	0.00479	0.00848	19.74999	
KD2	176	0.25436	0.82958	17.60282	41.3
KD3	204	0.12011	1.08487	15.65517	44.7
KD4	250	0.87397	1.44122	13.88335	17.2
KD5	312	1.23520	3.38453	11.93809	30.1
KD6	358	4.43288	4.77529	11.34784	110.9
KD7	443	2.37298	3.11198	12.06285	83.3
KD8	475	1.12223	5.86668	14.92877	83.0

[a] The angle between the principal magnetic axis of the given doublet and the that of the ground doublet.

Table S9. Properties of the eight lowest KDs of the Dy2 ion in **1** corresponding to the crystal-field split states in the ground $^6H_{15/2}$ multiplet.

KD	E / cm^{-1}	g_x	g_y	g_z	θ / ° [a]
KD1	0	0.00620	0.01058	19.75185	
KD2	181	0.43292	1.36588	17.74407	43.0
KD3	210	0.16902	1.93981	14.94068	42.6
KD4	245	0.63548	1.76575	13.50643	18.6
KD5	308	1.27063	3.41690	12.06350	32.7
KD6	362	4.01888	4.92099	10.98782	109.6
KD7	447	1.67094	2.54136	12.66437	83.6
KD8	484	1.02722	4.73611	15.90613	82.9

[a] The angle between the principal magnetic axis of the given doublet and the that of the ground doublet.

Table S10. Properties of the eight lowest KDs of the Dy1 ion in **2** corresponding to the crystal-field split states in the ground $^6H_{15/2}$ multiplet.

KD	E / cm^{-1}	g_x	g_y	g_z	θ / ° [a]
KD1	0	0.00274	0.00399	19.73043	
KD2	214	0.03671	0.04357	17.03462	4.5
KD3	364	0.30682	0.35781	14.55476	12.7
KD4	452	2.66307	3.29566	10.58803	11.7
KD5	504	2.13873	4.13363	11.50150	100.5
KD6	523	2.05568	3.44784	14.26742	91.1
KD7	606	0.41509	0.51420	16.45785	88.4
KD8	742	0.00248	0.02175	19.41816	89.4

[a] The angle between the principal magnetic axis of the given doublet and the that of the ground doublet.

Table S11. Properties of the eight lowest KDs of the Dy2 ion in **2** corresponding to the crystal-field split states in the ground $^6H_{15/2}$ multiplet.

KD	E / cm^{-1}	g_x	g_y	g_z	θ / ° [a]
KD1	0	0.00218	0.00299	19.72401	
KD2	208	0.02531	0.02897	17.08756	4.2
KD3	362	0.31294	0.40615	14.60738	12.7
KD4	457	2.29997	2.49618	10.74650	3.6
KD5	523	4.59049	5.79729	8.26972	100.2
KD6	574	1.08694	3.34816	11.85330	91.2
KD7	606	1.70246	4.35865	14.29206	81.8
KD8	717	0.01208	0.06229	19.31908	88.7

[a] The angle between the principal magnetic axis of the given doublet and the that of the ground doublet.

Table S12. Properties of the eight lowest KDs of the Dy1 ion in **3** corresponding to the crystal-field split states in the ground $^6H_{15/2}$ multiplet.

KD	E / cm^{-1}	g_x	g_y	g_z	θ / ° [a]
KD1	0	0.00088	0.00125	19.79452	
KD2	336	0.03341	0.04471	16.86470	2.4
KD3	546	0.58986	0.81103	13.87933	3.9
KD4	667	3.87667	4.83694	9.23525	4.5
KD5	754	3.37642	4.60129	9.14094	89.4
KD6	860	0.19052	0.51856	13.14480	90.3
KD7	996	0.10953	0.19171	16.44215	90.5
KD8	1223	0.00842	0.01449	19.55260	90.1

[a] The angle between the principal magnetic axis of the given doublet and the that of the ground doublet.

Table S13. Properties of the eight lowest KDs of the Dy2 ion in **3** corresponding to the crystal-field split states in the ground $^6H_{15/2}$ multiplet.

KD	E / cm^{-1}	g_x	g_y	g_z	θ / ° [a]
KD1	0	0.00149	0.00216	19.76203	
KD2	339	0.05755	0.07880	16.79071	2.7
KD3	551	0.95515	1.34018	13.64853	5.0
KD4	666	7.70619	6.76726	5.06887	92.8
KD5	749	1.38889	3.01956	10.24814	87.0
KD6	862	0.37624	0.48478	13.46090	88.6
KD7	1015	0.15325	0.24826	16.52331	90.1
KD8	1274	0.00829	0.01401	19.55805	90.5

[a] The angle between the principal magnetic axis of the given doublet and the that of the ground doublet.

Table S14. Magnitudes of transition magnetic moment matrix elements (in units Bohr magneton) calculated for the local transitions at the ions Dy1 and Dy2 in **1**.

		Dy1		Dy2	
Inital KD	Final KD	Climbing transition	Crossing transition	Climbing transition	Crossing transition
KD1	KD1	3.291665	0.002212	3.291975	0.002796
KD1	KD2	1.191059	0.029343	1.161377	0.056041
KD1	KD3	0.768153	0.065199	0.842669	0.142697
KD1	KD4	1.262320	0.058316	1.214816	0.087898
KD1	KD5	0.394280	0.071236	0.398014	0.070753
KD1	KD6	0.101675	0.040579	0.122881	0.049457
KD1	KD7	0.096774	0.051379	0.098209	0.052520
KD1	KD8	0.067628	0.016167	0.068360	0.016895
KD2	KD2	4.768655	0.222249	4.738430	0.372267
KD2	KD3	1.778078	0.411578	1.712467	0.745717
KD2	KD4	1.121237	0.246331	0.978310	0.417079
KD2	KD5	1.130502	0.135065	1.175628	0.171588
KD2	KD6	0.649182	0.370377	0.614733	0.366946
KD2	KD7	0.412373	0.176396	0.416443	0.178843
KD2	KD8	0.343610	0.069167	0.304390	0.080474
KD3	KD3	3.907043	0.307230	3.755350	0.566884
KD3	KD4	2.267619	0.197630	2.496346	0.371986
KD3	KD5	1.008769	0.365734	1.128982	0.488290
KD3	KD6	0.887476	0.505609	0.806077	0.500730
KD3	KD7	0.654899	0.178468	0.625540	0.135222
KD3	KD8	0.514433	0.140311	0.522256	0.121337
KD4	KD4	3.122653	0.396593	2.901285	0.429351
KD4	KD5	2.560165	0.511232	2.469863	0.569172
KD4	KD6	1.146126	0.236643	1.203894	0.301678
KD4	KD7	0.340957	0.412773	0.368513	0.390112

KD4	KD8	0.289573	0.307874	0.299717	0.339233
KD5	KD5	2.864311	0.863675	2.798329	0.891733
KD5	KD6	2.849591	0.912419	2.871937	0.826934
KD5	KD7	1.042033	0.674045	0.928482	0.680364
KD5	KD8	0.417125	0.462054	0.347003	0.441763
KD6	KD6	2.410792	2.351807	2.208671	2.345720
KD6	KD7	2.218937	1.373099	2.386844	1.342227
KD6	KD8	0.481712	0.801825	0.487966	0.758505
KD7	KD7	1.536610	2.254994	1.512248	2.163148
KD7	KD8	2.758754	1.431747	2.621463	1.404992
KD8	KD8	2.933496	2.306496	3.268990	2.059900

Table S15. Magnitudes of transition magnetic moment matrix elements (in units Bohr magneton) calculated for the local transitions at the ions Dy1 and Dy2 in **2**.

		Dy1		Dy2	
Inital KD	Final KD	Climbing transition	Crossing transition	Climbing transition	Crossing transition
KD1	KD1	3.288405	0.001123	3.287335	0.000861
KD1	KD2	1.765942	0.002747	1.755412	0.001843
KD1	KD3	0.361743	0.006550	0.387058	0.004550
KD1	KD4	0.251945	0.020082	0.292724	0.015904
KD1	KD5	0.105840	0.059938	0.107967	0.078889
KD1	KD6	0.032236	0.029046	0.048934	0.071133
KD1	KD7	0.035335	0.032379	0.048019	0.017668
KD1	KD8	0.006205	0.009610	0.018723	0.013927
KD2	KD2	3.145866	0.013430	3.085616	0.009078
KD2	KD3	2.339174	0.025605	2.277904	0.017794
KD2	KD4	0.519109	0.096065	0.529291	0.075758
KD2	KD5	0.232829	0.151772	0.286651	0.104190
KD2	KD6	0.207686	0.079254	0.272949	0.029112
KD2	KD7	0.062795	0.121772	0.123227	0.066496
KD2	KD8	0.034481	0.057803	0.060868	0.058648
KD3	KD3	3.105030	0.112677	2.975655	0.122107
KD3	KD4	2.847243	0.171326	2.829907	0.174910
KD3	KD5	0.500956	0.471373	0.325563	0.421889
KD3	KD6	0.258846	0.199053	0.215095	0.175225
KD3	KD7	0.083297	0.164888	0.138884	0.123154
KD3	KD8	0.054878	0.142586	0.097490	0.102813
KD4	KD4	2.161205	1.007396	1.938299	0.800777
KD4	KD5	2.852985	0.951273	2.992611	0.666717
KD4	KD6	1.383939	0.813802	1.134434	0.571026
KD4	KD7	0.338513	0.780254	0.249250	0.492120

KD4	KD8	0.097364	0.252921	0.153493	0.257842
KD5	KD5	1.794809	2.151969	1.104709	2.303059
KD5	KD6	2.671328	1.373673	2.329286	1.999268
KD5	KD7	0.967197	1.152285	1.259938	0.774334
KD5	KD8	0.224366	0.298115	0.336965	0.375015
KD6	KD6	0.829617	3.356409	0.912897	2.717044
KD6	KD7	1.607646	1.234361	2.423418	1.673517
KD6	KD8	0.264879	0.215390	0.534583	0.815489
KD7	KD7	2.473152	2.190276	2.941035	2.399021
KD7	KD8	1.738332	0.676618	1.694144	0.659125
KD8	KD8	3.447129	0.045485	3.954984	0.501585

Table S16. Magnitudes of transition magnetic moment matrix elements (in units Bohr magneton) calculated for the local transitions at the ions Dy1 and Dy2 in **3**.

		Dy1		Dy2	
Inital KD	Final KD	Climbing transition	Crossing transition	Climbing transition	Crossing transition
KD1	KD1	3.299087	0.000355	3.293671	0.000608
KD1	KD2	1.778952	0.001047	1.789823	0.002015
KD1	KD3	0.203014	0.006290	0.237074	0.009976
KD1	KD4	0.127356	0.011571	0.085140	0.023305
KD1	KD5	0.022207	0.063098	0.035425	0.064478
KD1	KD6	0.049263	0.024224	0.029378	0.047591
KD1	KD7	0.017793	0.014819	0.022419	0.014036
KD1	KD8	0.003261	0.006302	0.003926	0.005515
KD2	KD2	2.938051	0.013050	2.970129	0.022758
KD2	KD3	2.396219	0.022989	2.399274	0.046114
KD2	KD4	0.282524	0.132999	0.335837	0.199881
KD2	KD5	0.219545	0.052921	0.161266	0.098088
KD2	KD6	0.050480	0.138382	0.123116	0.038013
KD2	KD7	0.036002	0.082605	0.032783	0.087626
KD2	KD8	0.017704	0.027211	0.021988	0.029492
KD3	KD3	2.497845	0.235179	2.533654	0.384248
KD3	KD4	2.790721	0.275968	2.705321	0.435812
KD3	KD5	0.320310	0.673842	0.436690	0.914725
KD3	KD6	0.245950	0.113135	0.114054	0.241136
KD3	KD7	0.057973	0.045420	0.064721	0.071834
KD3	KD8	0.015556	0.047344	0.031261	0.057295
KD4	KD4	1.650281	1.456105	1.382180	1.986458
KD4	KD5	2.726045	0.884363	2.507808	1.440972
KD4	KD6	0.385336	0.794635	0.693880	0.368283
KD4	KD7	0.127760	0.198580	0.112796	0.143454

KD4	KD8	0.043918	0.047793	0.059857	0.055435
KD5	KD5	0.608477	2.300241	0.876379	2.096863
KD5	KD6	2.208977	1.462988	1.890135	1.810716
KD5	KD7	0.489370	0.272382	0.407479	0.261066
KD5	KD8	0.106746	0.120846	0.060565	0.086747
KD6	KD6	0.852099	2.443702	1.431970	1.993935
KD6	KD7	1.901331	1.657055	1.744452	1.470412
KD6	KD8	0.416037	0.244819	0.209215	0.387146
KD7	KD7	2.658463	2.020018	0.677494	3.184922
KD7	KD8	1.731233	0.528277	1.413467	1.136511
KD8	KD8	3.431142	0.672713	3.469250	0.155045

Table S17. Ab initio CF parameters (in cm^{-1}) calculated for ions Dy1 and Dy2 in **1** given in the Iwahara–Chibotaru notation.

		Dy1			Dy2		
k	q	Re(B_{kq})	Im(B_{kq})	$\lvert B_{kq}\rvert$	Re(B_{kq})	Im(B_{kq})	$\lvert B_{kq}\rvert$
2	0	−217.864787	−0.000000	217.864787	−221.947066	0.000000	221.947066
2	1	−2.944867	15.569181	15.845241	8.201500	16.530780	18.453490
2	2	57.749647	−10.919691	58.772965	56.130004	5.050304	56.356747
4	0	−15.280894	−0.000000	15.280894	−13.119309	0.000000	13.119309
4	1	9.330413	−0.399073	9.338944	−10.621130	−0.327433	10.626176
4	2	−0.800377	3.455929	3.547401	−4.816016	−4.507403	6.596264
4	3	−22.826152	15.550145	27.619563	27.951691	10.989485	30.034411
4	4	−20.002480	12.058383	23.356023	−21.695340	−5.581631	22.401839
6	0	−40.181623	−0.000000	40.181623	−40.347792	0.000000	40.347792
6	1	−5.933960	−5.674241	8.210291	4.482989	−6.070611	7.546490
6	2	−18.205277	1.652452	18.280118	−17.915164	1.274549	17.960445
6	3	−1.739831	−2.630103	3.153482	−1.034388	−3.289221	3.448033
6	4	0.783479	1.433999	1.634073	2.072750	−1.488023	2.551568
6	5	10.846429	−14.161311	17.837817	−14.459379	−10.406803	17.815027
6	6	−1.196002	9.212054	9.289368	−2.375379	−9.000646	9.308816
8	0	0.214922	−0.000000	0.214922	0.187375	0.000000	0.187375
8	1	0.006046	−0.012321	0.013725	0.021491	−0.017117	0.027474
8	2	0.102007	−0.026732	0.105452	0.111377	0.014263	0.112286
8	3	0.003755	0.054809	0.054938	0.043130	0.055690	0.070438
8	4	−0.026497	−0.004543	0.026884	−0.030844	0.021177	0.037414
8	5	−0.046127	0.078247	0.090831	0.072267	0.066528	0.098226
8	6	−0.002400	−0.010213	0.010491	0.001491	0.013257	0.013341
8	7	−0.010010	0.003087	0.010475	0.009877	0.000310	0.009882
8	8	−0.003766	0.057514	0.057637	−0.025260	−0.051410	0.057280
10	0	0.015597	0.000000	0.015597	0.018327	0.000000	0.018327

10	1	0.008813	−0.011912	0.014818	−0.009011	−0.009195	0.012874
10	2	0.003358	0.004362	0.005504	0.004915	−0.003335	0.005940
10	3	0.010711	−0.006540	0.012550	−0.013105	−0.003706	0.013619
10	4	−0.001587	−0.006794	0.006977	−0.000588	0.006942	0.006967
10	5	−0.009809	0.008700	0.013111	0.009260	0.005653	0.010849
10	6	−0.003566	−0.005909	0.006902	−0.003894	0.006711	0.007759
10	7	−0.001740	0.004713	0.005024	0.006306	0.004640	0.007829
10	8	−0.001691	0.007781	0.007963	−0.007761	−0.006244	0.009961
10	9	−0.003673	0.005647	0.006736	0.007667	0.002425	0.008041
10	10	−0.002122	−0.002438	0.003232	−0.001824	0.002578	0.003158
12	0	0.006661	0.000000	0.006661	0.006662	−0.000000	0.006662
12	1	0.000713	0.004106	0.004167	−0.000037	0.004154	0.004154
12	2	0.005161	−0.000807	0.005224	0.004981	0.000001	0.004981
12	3	0.000250	0.001658	0.001676	0.000569	0.001608	0.001705
12	4	0.000628	−0.001487	0.001615	0.000526	0.001130	0.001246
12	5	−0.000791	0.001801	0.001967	0.001435	0.001382	0.001993
12	6	0.000671	−0.000471	0.000820	0.000647	0.000211	0.000681
12	7	−0.000108	0.000018	0.000110	0.000068	−0.000029	0.000074
12	8	−0.000094	0.000015	0.000095	0.000020	0.000085	0.000087
12	9	0.000162	−0.000052	0.000170	−0.000255	−0.000023	0.000256
12	10	−0.000227	−0.000758	0.000791	0.000227	0.000704	0.000739
12	11	0.000524	0.000509	0.000730	−0.000269	0.000680	0.000731
12	12	−0.000297	−0.000121	0.000321	−0.000237	0.000188	0.000303
14	0	0.000009	−0.000000	0.000009	0.000006	−0.000000	0.000006
14	1	−0.000004	−0.000003	0.000005	0.000004	−0.000002	0.000005
14	2	−0.000016	0.000000	0.000016	−0.000016	0.000001	0.000016
14	3	−0.000002	−0.000005	0.000006	0.000000	−0.000005	0.000005
14	4	−0.000009	0.000000	0.000009	−0.000008	0.000003	0.000009
14	5	−0.000002	−0.000001	0.000002	0.000001	−0.000001	0.000002

14	6	0.000002	0.000001	0.000002	0.000002	−0.000002	0.000002
14	7	−0.000001	0.000001	0.000002	0.000000	0.000001	0.000001
14	8	−0.000001	0.000001	0.000001	−0.000000	−0.000001	0.000001
14	9	−0.000001	−0.000001	0.000001	0.000001	−0.000001	0.000001
14	10	0.000001	0.000002	0.000003	0.000000	−0.000002	0.000002
14	11	−0.000002	−0.000002	0.000002	0.000000	−0.000002	0.000002
14	12	0.000001	−0.000000	0.000001	0.000001	0.000000	0.000001
14	13	−0.000001	−0.000001	0.000001	−0.000000	−0.000001	0.000001
14	14	0.000000	−0.000000	0.000000	0.000001	0.000001	0.000001

[a] The CF parameters are only listed for non-negative values of q. The values with negative q are given by $B_{k-q} = (-1)^q B_{kq}{}^*$.

Table S18. Ab initio CF parameters (in units cm^{-1}) calculated for ions Dy1 and Dy2 in **2** given in the Iwahara–Chibotaru notation.

		Dy1			Dy2		
k	q	$Re(B_{kq})$	$Im(B_{kq})$	$\lvert B_{kq}\rvert$	$Re(B_{kq})$	$Im(B_{kq})$	$\lvert B_{kq}\rvert$
2	0	−375.174477	0.000000	375.174477	−384.524599	0.000000	384.524599
2	1	14.873986	10.466591	18.187495	−21.269855	4.763174	21.796664
2	2	78.638856	−22.171974	81.704749	38.571337	−43.223953	57.931495
4	0	−33.791047	0.000000	33.791047	−33.560626	0.000000	33.560626
4	1	−3.500553	−2.766958	4.462054	5.599025	−0.985325	5.685063
4	2	−2.013025	2.049238	2.872567	3.874947	−1.764794	4.257900
4	3	−2.986432	6.740336	7.372306	4.734381	−2.554609	5.379628
4	4	11.465400	1.118393	11.519817	10.137586	−4.052503	10.917575
6	0	−6.034260	0.000000	6.034260	−1.580183	0.000000	1.580183
6	1	−2.623946	−1.880810	3.228396	3.505852	−1.705604	3.898729
6	2	23.392040	6.143273	24.185271	26.308759	1.946765	26.380688
6	3	0.079383	2.901127	2.902213	−1.200737	−0.648553	1.364694
6	4	−0.579135	−2.393620	2.462684	−0.875940	−4.009266	4.103837
6	5	2.911424	−0.285762	2.925414	0.433991	−0.012446	0.434169
6	6	−8.016974	2.700369	8.459543	−8.302768	−2.992618	8.825629
8	0	0.289864	0.000000	0.289864	0.114736	0.000000	0.114736
8	1	0.036262	0.031088	0.047764	−0.075361	0.084768	0.113424
8	2	−0.566848	−0.132648	0.582162	−0.639976	−0.038013	0.641104
8	3	0.025325	−0.036657	0.044555	0.021121	0.026462	0.033857
8	4	−0.049759	0.028181	0.057185	−0.017690	0.080386	0.082309
8	5	−0.017869	−0.005568	0.018716	−0.014456	−0.002766	0.014718
8	6	0.017971	−0.016646	0.024496	0.015916	−0.000924	0.015943
8	7	0.006564	−0.000875	0.006622	0.002640	−0.001351	0.002965
8	8	0.001102	0.009312	0.009377	0.005427	0.014817	0.015779
10	0	−0.003873	−0.000000	0.003873	0.000906	−0.000000	0.000906

10	1	0.006611	0.006174	0.009046	−0.007866	0.000803	0.007907
10	2	−0.024709	−0.009564	0.026496	−0.031776	−0.005963	0.032330
10	3	0.000853	−0.006854	0.006907	0.002190	0.001579	0.002700
10	4	−0.006741	0.001417	0.006888	−0.005554	0.001340	0.005713
10	5	−0.002824	0.001454	0.003177	0.000869	0.000116	0.000876
10	6	0.002738	−0.000894	0.002880	0.002110	0.000515	0.002172
10	7	0.002642	0.001060	0.002847	−0.001115	−0.000382	0.001179
10	8	−0.007113	0.000343	0.007121	−0.007823	−0.003815	0.008704
10	9	0.000905	−0.001903	0.002107	−0.000706	0.000446	0.000835
10	10	−0.003449	0.001228	0.003661	−0.003642	0.000803	0.003729
12	0	0.006092	0.000000	0.006092	0.007340	−0.000000	0.007340
12	1	0.001468	0.000062	0.001469	−0.001638	−0.000979	0.001909
12	2	−0.001044	0.000028	0.001045	−0.000229	−0.000333	0.000405
12	3	−0.000133	−0.000318	0.000345	0.000192	−0.000016	0.000192
12	4	0.001155	0.000809	0.001410	0.001430	0.000169	0.001440
12	5	−0.000102	0.000339	0.000354	−0.000012	0.000051	0.000053
12	6	0.000050	−0.000159	0.000166	0.000004	−0.000226	0.000226
12	7	−0.000075	−0.000053	0.000092	0.000107	0.000008	0.000108
12	8	0.000173	0.000018	0.000174	0.000159	0.000123	0.000201
12	9	−0.000030	−0.000044	0.000053	0.000005	−0.000004	0.000007
12	10	0.000087	0.000029	0.000092	0.000021	0.000084	0.000086
12	11	−0.000095	0.000052	0.000108	−0.000013	−0.000002	0.000013
12	12	0.000175	−0.000136	0.000222	0.000185	0.000157	0.000243
14	0	−0.000009	−0.000000	0.000009	−0.000011	−0.000000	0.000011
14	1	−0.000001	−0.000003	0.000004	0.000000	0.000001	0.000001
14	2	0.000002	0.000002	0.000003	0.000001	0.000005	0.000005
14	3	−0.000002	0.000006	0.000006	−0.000000	−0.000003	0.000003
14	4	0.000001	−0.000003	0.000003	0.000001	−0.000003	0.000003
14	5	0.000002	−0.000003	0.000004	−0.000001	0.000000	0.000001

14	6	−0.000002	0.000003	0.000003	−0.000002	0.000002	0.000002
14	7	0.000000	0.000000	0.000000	−0.000001	−0.000001	0.000001
14	8	0.000001	−0.000000	0.000001	0.000001	0.000000	0.000001
14	9	−0.000000	0.000001	0.000001	−0.000000	−0.000000	0.000000
14	10	−0.000000	−0.000001	0.000001	−0.000000	−0.000001	0.000001
14	11	0.000000	−0.000000	0.000000	−0.000000	−0.000000	0.000000
14	12	−0.000000	0.000000	0.000000	−0.000000	−0.000000	0.000000
14	13	−0.000000	−0.000000	0.000000	0.000000	0.000000	0.000000
14	14	0.000000	−0.000000	0.000000	0.000000	0.000000	0.000000

a The CF parameters are only listed for non-negative values of q. The values with negative q are given by $B_{k-q} = (-1)^q B_{kq}{}^*$.

Table S19. Ab initio CF parameters (in units cm^{-1}) calculated for ions Dy1 and Dy2 in **3** given in the Iwahara–Chibotaru notation.

		Dy1			Dy2		
k	q	Re(B_{kq})	Im(B_{kq})	$\|B_{kq}\|$	Re(B_{kq})	Im(B_{kq})	$\|B_{kq}\|$
2	0	−608.510593	0.000000	608.510593	−615.722105	−0.000000	615.722105
2	1	−0.256494	−7.827711	7.831912	3.767179	7.907814	8.759290
2	2	172.214482	−46.227673	178.311036	199.557347	−37.906901	203.125744
4	0	−35.784616	0.000000	35.784616	−31.883265	0.000000	31.883265
4	1	−2.597748	3.212902	4.131710	5.231385	−2.077651	5.628857
4	2	−4.625638	2.742312	5.377434	−5.783541	2.929790	6.483287
4	3	−4.805053	6.543613	8.118338	4.351116	−3.766872	5.755131
4	4	−3.558383	4.487639	5.727215	−5.008925	5.419912	7.380026
6	0	−19.373989	0.000000	19.373989	−22.102600	0.000000	22.102600
6	1	1.081495	1.902326	2.188259	−3.582650	−2.299633	4.257193
6	2	18.875825	11.988023	22.360891	23.125446	6.648760	24.062258
6	3	−1.718678	0.448793	1.776308	2.718959	0.861218	2.852093
6	4	0.061843	−0.544617	0.548117	1.530063	2.673076	3.080005
6	5	−2.856283	0.075271	2.857275	2.592494	−1.197367	2.855646
6	6	6.179880	−1.335932	6.322629	7.947518	2.838255	8.439119
8	0	0.751164	0.000000	0.751164	1.009172	0.000000	1.009172
8	1	−0.036413	−0.088440	0.095643	0.174961	0.077396	0.191315
8	2	−0.738245	−0.367219	0.824533	−0.929473	−0.183699	0.947452
8	3	0.023104	−0.034747	0.041727	−0.001914	0.004149	0.004569
8	4	−0.112121	−0.012808	0.112850	−0.185950	−0.066258	0.197402
8	5	0.039425	−0.006112	0.039896	−0.033882	0.021439	0.040095
8	6	−0.023351	0.010811	0.025732	−0.032107	−0.007010	0.032864
8	7	−0.012017	0.000608	0.012032	0.015336	−0.003691	0.015773
8	8	0.014702	−0.013460	0.019933	0.028514	−0.000404	0.028517
10	0	0.016119	−0.000000	0.016119	0.013545	0.000000	0.013545

10	1	0.002814	0.002645	0.003862	−0.010307	−0.000296	0.010311
10	2	−0.023181	−0.020100	0.030682	−0.026255	−0.011364	0.028609
10	3	0.000979	−0.003134	0.003283	−0.002368	0.000192	0.002376
10	4	0.000073	−0.005408	0.005409	−0.000472	−0.006752	0.006769
10	5	0.002275	0.001894	0.002961	−0.002669	−0.000913	0.002821
10	6	−0.004964	−0.000608	0.005002	−0.005914	−0.002344	0.006361
10	7	−0.000818	−0.000650	0.001044	0.000970	−0.001062	0.001438
10	8	0.004958	0.001282	0.005121	0.005140	0.003125	0.006016
10	9	−0.000118	0.000770	0.000779	0.000804	0.000524	0.000960
10	10	−0.000744	0.001335	0.001528	−0.002197	0.001713	0.002786
12	0	0.006416	0.000000	0.006416	0.007214	0.000000	0.007214
12	1	−0.001126	0.000390	0.001192	0.002772	−0.000253	0.002783
12	2	−0.002905	−0.001183	0.003137	−0.003163	−0.000471	0.003198
12	3	−0.000012	0.000043	0.000045	−0.000219	−0.000199	0.000296
12	4	0.000664	0.001151	0.001329	0.001189	0.000657	0.001359
12	5	−0.000046	−0.000115	0.000124	0.000185	0.000257	0.000317
12	6	0.000064	0.000255	0.000263	0.000118	0.000281	0.000305
12	7	−0.000036	−0.000025	0.000044	0.000067	0.000161	0.000174
12	8	−0.000119	−0.000078	0.000142	−0.000110	−0.000110	0.000155
12	9	−0.000004	0.000011	0.000011	0.000044	0.000043	0.000061
12	10	−0.000028	−0.000017	0.000033	−0.000047	0.000002	0.000047
12	11	−0.000064	0.000014	0.000066	0.000088	−0.000008	0.000088
12	12	0.000109	−0.000045	0.000118	0.000157	0.000132	0.000205
14	0	−0.000022	−0.000000	0.000022	−0.000034	−0.000000	0.000034
14	1	0.000007	−0.000004	0.000008	−0.000020	0.000004	0.000020
14	2	0.000010	0.000003	0.000011	0.000014	−0.000001	0.000014
14	3	0.000002	−0.000001	0.000003	−0.000004	0.000003	0.000005
14	4	0.000001	−0.000001	0.000001	−0.000001	0.000000	0.000001
14	5	−0.000001	0.000001	0.000002	−0.000000	−0.000003	0.000003

14	6	0.000002	−0.000001	0.000003	0.000005	0.000002	0.000005
14	7	0.000001	−0.000001	0.000001	−0.000001	0.000001	0.000002
14	8	−0.000001	0.000000	0.000001	−0.000001	−0.000001	0.000002
14	9	−0.000000	0.000000	0.000000	−0.000000	−0.000000	0.000000
14	10	0.000000	0.000000	0.000000	0.000000	0.000000	0.000000
14	11	0.000000	0.000000	0.000000	−0.000000	−0.000000	0.000000
14	12	−0.000000	0.000000	0.000000	−0.000000	−0.000000	0.000001
14	13	−0.000000	0.000000	0.000000	0.000000	−0.000000	0.000000
14	14	0.000000	−0.000000	0.000000	0.000000	0.000000	0.000000

a The CF parameters are only listed for non-negative values of q. The values with negative q are given by $B_{k-q} = (-1)^q B_{kq}{}^*$.

Table S20. Squared magnitudes of projections of the ab initio CF eigenstates calculated for the Dy1 ion in **1** onto angular momentum eigenstates with angular momentum $J = 15/2$ and projection M.

M	KD1		KD2		KD3		KD4		KD5		KD6		KD7		KD8	
−15/2	0.975	0.008	0.000	0.005	0.000	0.001	0.000	0.005	0.003	0.000	0.000	0.002	0.000	0.000	0.000	0.000
−13/2	0.000	0.000	0.034	0.311	0.016	0.146	0.027	0.387	0.063	0.005	0.003	0.000	0.002	0.001	0.003	0.001
−11/2	0.007	0.000	0.029	0.214	0.003	0.176	0.001	0.123	0.310	0.001	0.003	0.101	0.013	0.009	0.006	0.003
−9/2	0.006	0.000	0.018	0.117	0.024	0.297	0.009	0.241	0.064	0.015	0.017	0.111	0.047	0.030	0.000	0.002
−7/2	0.000	0.000	0.020	0.180	0.001	0.117	0.002	0.105	0.398	0.015	0.018	0.013	0.090	0.010	0.025	0.007
−5/2	0.003	0.000	0.004	0.019	0.017	0.124	0.001	0.070	0.049	0.011	0.018	0.478	0.036	0.083	0.066	0.020
−3/2	0.000	0.000	0.006	0.033	0.003	0.031	0.005	0.007	0.034	0.016	0.140	0.061	0.471	0.046	0.116	0.032
−1/2	0.000	0.000	0.003	0.007	0.016	0.028	0.011	0.005	0.001	0.016	0.014	0.020	0.143	0.019	0.703	0.015
1/2	0.000	0.000	0.007	0.003	0.028	0.016	0.005	0.011	0.016	0.001	0.020	0.014	0.019	0.143	0.015	0.703
3/2	0.000	0.000	0.033	0.006	0.031	0.003	0.007	0.005	0.016	0.034	0.061	0.140	0.046	0.471	0.032	0.116
5/2	0.000	0.003	0.019	0.004	0.124	0.017	0.070	0.001	0.011	0.049	0.478	0.018	0.083	0.036	0.020	0.066
7/2	0.000	0.000	0.180	0.020	0.117	0.001	0.105	0.002	0.015	0.398	0.013	0.018	0.010	0.090	0.007	0.025
9/2	0.000	0.006	0.117	0.018	0.297	0.024	0.241	0.009	0.015	0.064	0.111	0.017	0.030	0.047	0.002	0.000
11/2	0.000	0.007	0.214	0.029	0.176	0.003	0.123	0.001	0.001	0.310	0.101	0.003	0.009	0.013	0.003	0.006
13/2	0.000	0.000	0.311	0.034	0.146	0.016	0.387	0.027	0.005	0.063	0.000	0.003	0.001	0.002	0.001	0.003
15/2	0.008	0.975	0.005	0.000	0.001	0.000	0.005	0.000	0.000	0.003	0.002	0.000	0.000	0.000	0.000	0.000

Table S21. Squared magnitudes of projections of the ab initio CF eigenstates calculated for the Dy2ion in **1** onto angular momentum eigenstates with angular momentum J = 15/2 and projection M.

M	KD1		KD2		KD3		KD4		KD5		KD6		KD7		KD8	
−15/2	0.025	0.957	0.000	0.006	0.000	0.000	0.006	0.000	0.000	0.002	0.002	0.000	0.000	0.000	0.000	0.000
−13/2	0.000	0.000	0.000	0.344	0.207	0.011	0.310	0.034	0.002	0.079	0.004	0.002	0.000	0.002	0.000	0.003
−11/2	0.000	0.010	0.006	0.239	0.151	0.006	0.161	0.003	0.002	0.277	0.087	0.027	0.020	0.004	0.000	0.005
−9/2	0.000	0.004	0.007	0.134	0.306	0.001	0.260	0.019	0.017	0.059	0.086	0.022	0.066	0.014	0.003	0.001
−7/2	0.000	0.000	0.003	0.184	0.116	0.004	0.094	0.000	0.008	0.432	0.015	0.014	0.064	0.027	0.019	0.021
−5/2	0.000	0.002	0.003	0.023	0.117	0.004	0.075	0.003	0.007	0.065	0.399	0.099	0.105	0.001	0.002	0.092
−3/2	0.000	0.000	0.000	0.038	0.030	0.002	0.011	0.005	0.010	0.026	0.132	0.080	0.059	0.432	0.029	0.144
−1/2	0.000	0.000	0.004	0.008	0.030	0.012	0.006	0.012	0.012	0.001	0.015	0.014	0.012	0.193	0.174	0.506
1/2	0.000	0.000	0.008	0.004	0.012	0.030	0.012	0.006	0.001	0.012	0.014	0.015	0.193	0.012	0.506	0.174
3/2	0.000	0.000	0.038	0.000	0.002	0.030	0.005	0.011	0.026	0.010	0.080	0.132	0.432	0.059	0.144	0.029
5/2	0.002	0.000	0.023	0.003	0.004	0.117	0.003	0.075	0.065	0.007	0.099	0.399	0.001	0.105	0.092	0.002
7/2	0.000	0.000	0.184	0.003	0.004	0.116	0.000	0.094	0.432	0.008	0.014	0.015	0.027	0.064	0.021	0.019
9/2	0.004	0.000	0.134	0.007	0.001	0.306	0.019	0.260	0.059	0.017	0.022	0.086	0.014	0.066	0.001	0.003
11/2	0.010	0.000	0.239	0.006	0.006	0.151	0.003	0.161	0.277	0.002	0.027	0.087	0.004	0.020	0.005	0.000
13/2	0.000	0.000	0.344	0.000	0.011	0.207	0.034	0.310	0.079	0.002	0.002	0.004	0.002	0.000	0.003	0.000
15/2	0.957	0.025	0.006	0.000	0.000	0.000	0.000	0.006	0.002	0.000	0.000	0.002	0.000	0.000	0.000	0.000

Table S22. Squared magnitudes of projections of the ab initio CF eigenstates calculated for the Dy1 ion in **2** onto angular momentum eigenstates with angular momentum J = 15/2 and projection M.

M	KD1		KD2		KD3		KD4		KD5		KD6		KD7		KD8	
−15/2	0.302	0.668	0.000	0.000	0.016	0.010	0.000	0.003	0.000	0.000	0.000	0.000	0.000	0.000	0.000	0.000
−13/2	0.000	0.000	0.928	0.038	0.006	0.004	0.001	0.018	0.003	0.001	0.000	0.001	0.000	0.000	0.000	0.000
−11/2	0.009	0.020	0.008	0.000	0.549	0.325	0.000	0.071	0.004	0.006	0.004	0.001	0.002	0.001	0.000	0.000
−9/2	0.000	0.000	0.022	0.001	0.036	0.017	0.023	0.686	0.129	0.043	0.000	0.014	0.006	0.021	0.000	0.000
−7/2	0.000	0.000	0.001	0.000	0.014	0.009	0.008	0.058	0.279	0.040	0.340	0.059	0.141	0.036	0.006	0.009
−5/2	0.000	0.000	0.000	0.000	0.004	0.002	0.003	0.060	0.045	0.052	0.077	0.197	0.052	0.391	0.088	0.029
−3/2	0.000	0.000	0.000	0.000	0.002	0.002	0.027	0.016	0.184	0.030	0.090	0.002	0.270	0.037	0.109	0.230
−1/2	0.000	0.000	0.001	0.000	0.002	0.004	0.004	0.023	0.050	0.132	0.048	0.167	0.011	0.030	0.375	0.153
1/2	0.000	0.000	0.000	0.001	0.004	0.002	0.023	0.004	0.132	0.050	0.167	0.048	0.030	0.011	0.153	0.375
3/2	0.000	0.000	0.000	0.000	0.002	0.002	0.016	0.027	0.030	0.184	0.002	0.090	0.037	0.270	0.230	0.109
5/2	0.000	0.000	0.000	0.000	0.002	0.004	0.060	0.003	0.052	0.045	0.197	0.077	0.391	0.052	0.029	0.088
7/2	0.000	0.000	0.000	0.001	0.009	0.014	0.058	0.008	0.040	0.279	0.059	0.340	0.036	0.141	0.009	0.006
9/2	0.000	0.000	0.001	0.022	0.017	0.036	0.686	0.023	0.043	0.129	0.014	0.000	0.021	0.006	0.000	0.000
11/2	0.020	0.009	0.000	0.008	0.325	0.549	0.071	0.000	0.006	0.004	0.001	0.004	0.001	0.002	0.000	0.000
13/2	0.000	0.000	0.038	0.928	0.004	0.006	0.018	0.001	0.001	0.003	0.001	0.000	0.000	0.000	0.000	0.000
15/2	0.668	0.302	0.000	0.000	0.010	0.016	0.003	0.000	0.000	0.000	0.000	0.000	0.000	0.000	0.000	0.000

Table S23. Squared magnitudes of projections of the ab initio CF eigenstates calculated for the Dy2 ion in **2** onto angular momentum eigenstates with angular momentum J = 15/2 and projection M.

M	KD1		KD2		KD3		KD4		KD5		KD6		KD7		KD8	
−15/2	0.562	0.407	0.000	0.000	0.001	0.027	0.001	0.000	0.001	0.000	0.000	0.000	0.000	0.000	0.000	0.000
−13/2	0.000	0.000	0.955	0.021	0.000	0.003	0.018	0.001	0.000	0.001	0.000	0.000	0.000	0.000	0.000	0.000
−11/2	0.018	0.013	0.007	0.000	0.038	0.841	0.054	0.001	0.020	0.001	0.004	0.001	0.000	0.002	0.000	0.001
−9/2	0.000	0.000	0.015	0.000	0.004	0.053	0.700	0.043	0.041	0.076	0.010	0.037	0.012	0.003	0.002	0.004
−7/2	0.000	0.000	0.001	0.000	0.001	0.023	0.056	0.007	0.488	0.006	0.343	0.006	0.031	0.018	0.005	0.014
−5/2	0.000	0.000	0.001	0.000	0.001	0.002	0.068	0.005	0.030	0.085	0.032	0.156	0.320	0.187	0.052	0.060
−3/2	0.000	0.000	0.000	0.000	0.000	0.004	0.008	0.014	0.126	0.012	0.078	0.065	0.159	0.192	0.131	0.212
−1/2	0.000	0.000	0.000	0.000	0.002	0.000	0.021	0.004	0.013	0.101	0.036	0.230	0.005	0.070	0.278	0.239
1/2	0.000	0.000	0.000	0.000	0.000	0.002	0.004	0.021	0.101	0.013	0.230	0.036	0.070	0.005	0.239	0.278
3/2	0.000	0.000	0.000	0.000	0.004	0.000	0.014	0.008	0.012	0.126	0.065	0.078	0.192	0.159	0.212	0.131
5/2	0.000	0.000	0.000	0.001	0.002	0.001	0.005	0.068	0.085	0.030	0.156	0.032	0.187	0.320	0.060	0.052
7/2	0.000	0.000	0.000	0.001	0.023	0.001	0.007	0.056	0.006	0.488	0.006	0.343	0.018	0.031	0.014	0.005
9/2	0.000	0.000	0.000	0.015	0.053	0.004	0.043	0.700	0.076	0.041	0.037	0.010	0.003	0.012	0.004	0.002
11/2	0.013	0.018	0.000	0.007	0.841	0.038	0.001	0.054	0.001	0.020	0.001	0.004	0.002	0.000	0.001	0.000
13/2	0.000	0.000	0.021	0.955	0.003	0.000	0.001	0.018	0.001	0.000	0.000	0.000	0.000	0.000	0.000	0.000
15/2	0.407	0.562	0.000	0.000	0.027	0.001	0.000	0.001	0.000	0.001	0.000	0.000	0.000	0.000	0.000	0.000

Table S24. Squared magnitudes of projections of the ab initio CF eigenstates calculated for the Dy1 ion in **3** onto angular momentum eigenstates with angular momentum J = 15/2 and projection M.

M	KD1		KD2		KD3		KD4		KD5		KD6		KD7		KD8	
−15/2	0.195	0.787	0.000	0.000	0.013	0.004	0.000	0.000	0.000	0.001	0.000	0.000	0.000	0.000	0.000	0.000
−13/2	0.000	0.000	0.906	0.050	0.003	0.001	0.018	0.016	0.004	0.001	0.001	0.001	0.000	0.000	0.000	0.000
−11/2	0.003	0.014	0.001	0.000	0.665	0.207	0.007	0.015	0.003	0.063	0.002	0.016	0.001	0.002	0.000	0.000
−9/2	0.000	0.000	0.038	0.002	0.004	0.001	0.349	0.300	0.150	0.015	0.097	0.013	0.023	0.004	0.004	0.000
−7/2	0.000	0.000	0.001	0.000	0.066	0.021	0.005	0.012	0.013	0.376	0.048	0.295	0.019	0.119	0.002	0.022
−5/2	0.000	0.000	0.001	0.000	0.002	0.000	0.098	0.083	0.016	0.002	0.254	0.046	0.320	0.060	0.104	0.011
−3/2	0.000	0.000	0.000	0.000	0.007	0.003	0.011	0.025	0.009	0.234	0.001	0.006	0.058	0.323	0.030	0.293
−1/2	0.000	0.000	0.000	0.000	0.002	0.002	0.034	0.025	0.106	0.008	0.190	0.030	0.062	0.009	0.482	0.050
1/2	0.000	0.000	0.000	0.000	0.002	0.002	0.025	0.034	0.008	0.106	0.030	0.190	0.009	0.062	0.050	0.482
3/2	0.000	0.000	0.000	0.000	0.003	0.007	0.025	0.011	0.234	0.009	0.006	0.001	0.323	0.058	0.293	0.030
5/2	0.000	0.000	0.000	0.001	0.000	0.002	0.083	0.098	0.002	0.016	0.046	0.254	0.060	0.320	0.011	0.104
7/2	0.000	0.000	0.000	0.001	0.021	0.066	0.012	0.005	0.376	0.013	0.295	0.048	0.119	0.019	0.022	0.002
9/2	0.000	0.000	0.002	0.038	0.001	0.004	0.300	0.349	0.015	0.150	0.013	0.097	0.004	0.023	0.000	0.004
11/2	0.014	0.003	0.000	0.001	0.207	0.665	0.015	0.007	0.063	0.003	0.016	0.002	0.002	0.001	0.000	0.000
13/2	0.000	0.000	0.050	0.906	0.001	0.003	0.016	0.018	0.001	0.004	0.001	0.001	0.000	0.000	0.000	0.000
15/2	0.787	0.195	0.000	0.000	0.004	0.013	0.000	0.000	0.001	0.000	0.000	0.000	0.000	0.000	0.000	0.000

Table S25. Squared magnitudes of projections of the ab initio CF eigenstates calculated for the Dy2 ion in **3** onto angular momentum eigenstates with angular momentum $J = 15/2$ and projection M.

M	KD1		KD2		KD3		KD4		KD5		KD6		KD7		KD8	
−15/2	0.821	0.155	0.000	0.000	0.001	0.020	0.000	0.001	0.001	0.000	0.000	0.000	0.000	0.000	0.000	0.000
−13/2	0.000	0.000	0.062	0.880	0.000	0.004	0.024	0.016	0.003	0.008	0.000	0.003	0.000	0.000	0.000	0.000
−11/2	0.019	0.004	0.000	0.002	0.056	0.779	0.003	0.038	0.065	0.005	0.024	0.001	0.003	0.001	0.000	0.001
−9/2	0.000	0.000	0.003	0.049	0.001	0.007	0.316	0.210	0.037	0.226	0.001	0.116	0.005	0.024	0.003	0.001
−7/2	0.000	0.000	0.000	0.000	0.007	0.097	0.015	0.014	0.274	0.036	0.380	0.007	0.120	0.026	0.006	0.018
−5/2	0.000	0.000	0.000	0.002	0.003	0.002	0.118	0.090	0.001	0.009	0.005	0.267	0.071	0.318	0.093	0.023
−3/2	0.000	0.000	0.000	0.000	0.001	0.015	0.025	0.038	0.205	0.020	0.004	0.000	0.301	0.066	0.083	0.241
−1/2	0.000	0.000	0.000	0.000	0.006	0.001	0.053	0.041	0.012	0.097	0.004	0.189	0.012	0.053	0.409	0.122
1/2	0.000	0.000	0.000	0.000	0.001	0.006	0.041	0.053	0.097	0.012	0.189	0.004	0.053	0.012	0.122	0.409
3/2	0.000	0.000	0.000	0.000	0.015	0.001	0.038	0.025	0.020	0.205	0.000	0.004	0.066	0.301	0.241	0.083
5/2	0.000	0.000	0.002	0.000	0.002	0.003	0.090	0.118	0.009	0.001	0.267	0.005	0.318	0.071	0.023	0.093
7/2	0.000	0.000	0.000	0.000	0.097	0.007	0.014	0.015	0.036	0.274	0.007	0.380	0.026	0.120	0.018	0.006
9/2	0.000	0.000	0.049	0.003	0.007	0.001	0.210	0.316	0.226	0.037	0.116	0.001	0.024	0.005	0.001	0.003
11/2	0.004	0.019	0.002	0.000	0.779	0.056	0.038	0.003	0.005	0.065	0.001	0.024	0.001	0.003	0.001	0.000
13/2	0.000	0.000	0.880	0.062	0.004	0.000	0.016	0.024	0.008	0.003	0.003	0.000	0.000	0.000	0.000	0.000
15/2	0.155	0.821	0.000	0.000	0.020	0.001	0.001	0.000	0.000	0.001	0.000	0.000	0.000	0.000	0.000	0.000

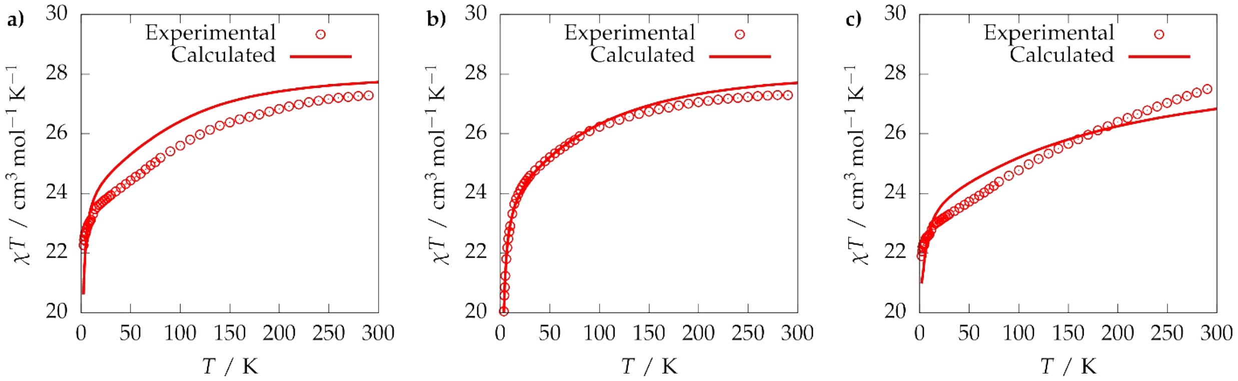

Fig. S28. Experimental and calculated magnetic susceptibility as a function of temperature, plotted as $\chi T(T)$, for **1** (a), **2** (b) and **3** (c).

References

1 R. Brand, H.-P. Krimmer, H.-J. Lindner, V. Sturm and K. Hafner, *Tetrahedron Lett.*, 1982, **23**, 5131–5134.

2 S. M. Cendrowski-Guillaume, G. Le Gland, M. Nierlich and M. Ephritikhine, *Organometallics*, 2000, **19**, 5654–5660.

3 S. J. Connelly, W. Kaminsky and D. M. Heinekey, *Organometallics*, 2013, **32**, 7478–7481.

4 O. V Dolomanov, L. J. Bourhis, R. J. Gildea, J. A. K. Howard and H. Puschmann, *J. Appl. Crystallogr.*, 2009, **42**, 339–341.

5 G. M. Sheldrick, *Acta Crystallogr. Sect. C*, 2015, **71**, 3–8.

6 G. M. Sheldrick, *Acta Crystallogr. Sect. A*, 2008, **64**, 112–122.

7 G. A. Bain and J. F. Berry, *J. Chem. Educ.*, 2008, **85**, 532.

8 G. te Velde, F. M. Bickelhaupt, E. J. Baerends, C. Fonseca Guerra, S. J. A. van Gisbergen, J. G. Snijders and T. Ziegler, *J. Comput. Chem.*, 2001, **22**, 931–967.

9 C. Fonseca Guerra, J. G. Snijders, G. te Velde and E. J. Baerends, *Theor. Chem. Acc.*, 1998, **99**, 391–403.

10 J. P. Perdew, K. Burke and M. Ernzerhof, *Phys. Rev. Lett.*, 1996, **77**, 3865–3868.

11 J. P. Perdew, K. Burke and M. Ernzerhof, *Phys. Rev. Lett.*, 1997, **78**, 1396.

12 S. Grimme, J. Antony, S. Ehrlich and H. Krieg, *J. Chem. Phys.*, 2010, **132**, 154104.

13 S. Grimme, S. Ehrlich and L. Goerigk, *J. Comput. Chem.*, 2011, **32**, 1456–1465.

14 E. van van Lenthe, E. J. Baerends and J. G. Snijders, *J. Chem. Phys.*, 1993, **99**, 4597–4610.

15 E. van Lenthe, E. J. Baerends and J. G. Snijders, *J. Chem. Phys.*, 1994, **101**, 9783–9792.

16 E. van Lenthe, R. van Leeuwen, E. J. Baerends and J. G. Snijders, *Int. J. Quantum Chem.*, 1996, **57**, 281–293.

17 E. van Lenthe and E. J. Baerends, *J. Comput. Chem.*, 2003, **24**, 1142–1156.

18 I. Fdez. Galván, M. Vacher, A. Alavi, C. Angeli, F. Aquilante, J. Autschbach, J. J. Bao, S. I. Bokarev, N. A. Bogdanov, R. K. Carlson, L. F. Chibotaru, J. Creutzberg, N. Dattani, M. G. Delcey, S. S. Dong, A. Dreuw, L. Freitag, L. M. Frutos, L. Gagliardi, F. Gendron, A. Giussani, L. González, G. Grell, M. Guo, C. E. Hoyer, M. Johansson, S. Keller, S. Knecht, G. Kovačević, E. Källman, G. Li Manni, M. Lundberg, Y. Ma, S. Mai, J. P. Malhado, P. Å. Malmqvist, P. Marquetand, S. A. Mewes, J. Norell, M. Olivucci, M. Oppel, Q. M. Phung, K. Pierloot, F. Plasser, M. Reiher, A. M. Sand, I. Schapiro, P. Sharma, C. J. Stein, L. K. Sørensen, D. G. Truhlar, M. Ugandi, L. Ungur, A. Valentini, S. Vancoillie, V. Veryazov, O. Weser, T. A. Wesołowski, P.-O. Widmark, S. Wouters, A. Zech, J. P. Zobel and R. Lindh, *J. Chem. Theory Comput.*, 2019, **15**, 5925–5964.

19 B. O. Roos, in *Advances in Chemical Physics: Ab Initio Methods in Quantum Chemistry II, Vol. 69*, ed. K. P. Lawley, Wiley, New York, NY, USA, 1987, pp. 399–455.

20 P. Siegbahn, A. Heiberg, B. Roos and B. Levy, *Phys. Scr.*, 1980, **21**, 323–327.

21 B. O. Roos, P. R. Taylor and P. E. M. Siegbahn, *Chem. Phys.*, 1980, **48**, 157–173.

22 P. E. M. Siegbahn, J. Almlöf, A. Heiberg and B. O. Roos, *J. Chem. Phys.*, 1981, **74**, 2384–2396.

23 B. O. Roos, R. Lindh, P. Å. Malmqvist, V. Veryazov and P.-O. Widmark, *Multiconfigurational Quantum Chemistry*, Wiley, Hoboken, NJ, USA, 2016.

24 P. Å. Malmqvist, B. O. Roos and B. Schimmelpfennig, *Chem. Phys. Lett.*, 2002, **357**, 230–240.

25 L. F. Chibotaru and L. Ungur, *J. Chem. Phys.*, 2012, **137**, 64112.

26 L. Ungur and L. F. Chibotaru, in *Lanthanides and Actinides in Molecular Magnetism*, eds. R. A. Layfield and M. Murugesu, Wiley-VHC, Weinheim, Germany, 2015, pp. 153–184.

27 L. F. Chibotaru, L. Ungur and A. Soncini, *Angew. Chemie Int. Ed.*, 2008, **47**, 4126–4129.

28 L. Ungur, W. den Heuvel and L. F. Chibotaru, *New J. Chem.*, 2009, **33**, 1224–1230.

29 M. E. Lines, *J. Chem. Phys.*, 1971, **55**, 2977–2984.

30 P.-O. Widmark, P.-Å. Malmqvist and B. O. Roos, *Theor. Chim. Acta*, 1990, **77**, 291–306.

31 B. O. Roos, R. Lindh, P.-Å. Malmqvist, V. Veryazov and and Per-Olof Widmark, *J. Phys. Chem. A*, 2004, **108**, 2851–2858.

32 Björn O. Roos, Roland Lindh, Per-Åke Malmqvist, Valera Veryazov and and Per-Olof Widmark, *J. Phys. Chem. A*, 2005, **109**, 6575–6579.

33 B. O. Roos, R. Lindh, P.-Å. Malmqvist, V. Veryazov, P.-O. Widmark and A. C. Borin, *J. Phys. Chem. A*, 2008, **112**, 11431–11435.

34 M. Filatov, *J. Chem. Phys.*, 2006, **125**, 107101.

35 W. Kutzelnigg and W. Liu, *J. Chem. Phys.*, 2005, **123**, 241102.

36 D. Peng and M. Reiher, *Theor. Chem. Acc.*, 2012, **131**, 1081.

37 B. A. Heß, C. M. Marian, U. Wahlgren and O. Gropen, *Chem. Phys. Lett.*, 1996, **251**, 365–371.
38 O. Christiansen, J. Gauss and B. Schimmelpfennig, *Phys. Chem. Chem. Phys.*, 2000, **2**, 965–971.
